# Metabolomic characterization benefits the identification of acute lung injury in patients with type A acute aortic dissection

**DOI:** 10.3389/fmolb.2023.1222133

**Published:** 2023-08-03

**Authors:** Linglin Fan, Ke Meng, Fanqi Meng, Yuan Wu, Ling Lin

**Affiliations:** ^1^ Xiamen Cardiovascular Hospital of Xiamen University, School of Medicine, Xiamen University, Xiamen, China; ^2^ Medical College, Guangxi University, Nanning, Guangxi, China; ^3^ Department of Cardiac Surgery, Yue Bei People’s Hospital, Shaoguan, Guangdong, China; ^4^ Shanghai Institute of Cardiovascular Diseases, Zhongshan Hospital, Fudan University, Shanghai, China

**Keywords:** acute lung injury, acute aortic dissection, targeted metabolomics, diagnostic panel, pathway enrichment analysis

## Abstract

**Introduction:** Acute aortic dissection (AAD) often leads to the development of acute lung injury (ALI). However, the early detection and diagnosis of AAD in patients with ALI pose significant challenges. The objective of this study is to investigate distinct metabolic alterations in the plasma samples of AAD patients with ALI, AAD patients without ALI, and healthy individuals.

**Method:** Between September 2019 and September 2022, we retrospectively collected data from 228 AAD patients who were diagnosed with ALI through post-surgery chest X-ray and PaO_2_/FiO_2_ assessments. Univariate analysis was employed to identify pre-surgery risk factors for ALI. Additionally, we conducted high-throughput target metabolic analysis on 90 plasma samples, comprising 30 samples from AAD patients with ALI, 30 from patients with AAD only, and 30 from healthy controls. After LC-MS spectral processing and metabolite quantification, the recursive feature elimination with cross-validation (RFECV) analysis based on the random forest was used to select the optimal metabolites as a diagnostic panel for the detection of AAD patients with ALI. The support vector machines (SVM) machine learning model was further applied to validate the diagnostic accuracy of the established biomarker panel.

**Results:** In the univariate analysis, preoperative β-HB and TNF-α exhibited a significant association with lung injury (OR = 0.906, 95% CI 0.852–0.965, *p* = 0.002; OR = 1.007, 95% CI 1.003–1.011, *p* < 0.0001). The multiple-reaction monitoring analysis of 417 common metabolites identified significant changes in 145 metabolites (fold change >1.2 or <0.833, *p* < 0.05) across the three groups. Multivariate statistical analysis revealed notable differences between AAD patients and healthy controls. When compared with the non-ALI group, AAD patients with ALI displayed remarkable upregulation in 19 metabolites and downregulation in 4 metabolites. Particularly, combining citric acid and glucuronic acid as a biomarker panel improved the classification performance for distinguishing between the ALI and non-ALI groups.

**Discussion:** Differentially expressed metabolites in the ALI group were primarily involved in amino acids biosynthesis, carbohydrate metabolism (TCA cycle), arginine and proline metabolism, and glucagon signaling pathway. These findings demonstrate a great potential of the targeted metabolomic approach for screening, routine surveillance, and diagnosis of pulmonary injury in patients with AAD.

## 1 Introduction

Acute type A aortic dissection (AAD) constitutes a cardiovascular emergency necessitating immediate intervention (Wang et al., 2022). Despite significant advancements in surgical techniques and postoperative care, the incidence of complications such as paraplegia, stroke, acute renal damage, low cardiac output syndrome, and acute lung injury (ALI) remains high (Wei et al., 2019). Worryingly, postoperative ALI has been reported in 30%–50% of AAD patients, which can lead to decreased oxygen levels in the blood and difficulty in breathing (Liu et al., 2016). In severe cases, ALI can progress to acute respiratory distress syndrome (ARDS), which is a life-threatening condition with a high mortality rate. It is important for clinicians to closely monitor patients for signs of ALI after surgery for AAD and to take appropriate measures to prevent this complication.

The mechanisms triggering the onset of postoperative ALI subsequent to AAD differ markedly and are distinct from those observed in general cardiac surgery with extracorporeal circulation. They encompass factors like rapid AAD onset, compromised thoracic organs’ blood supply, and an inability to establish collateral circulation (Girdauskas et al., 2010). Additionally, the aortic tear-induced inflammatory response and the subsequent release of inflammatory mediators, such as neutrophil elastase (NE), can inflict damage upon lung tissue (Gu et al., 2015). Other influential factors comprise the activation of the complement, kinin, and fibrinolytic coagulant systems during prolonged extracorporeal circulation (Roumy et al., 2020) along with ventilator-associated lung injury resulting from lung hyperinflation and excessive tissue stretching (Rich et al., 2000).

Clinical ALI diagnosis hinges on the criteria set forth by the 1994 European Consensus Criteria (Bernard et al., 1994) and the 2012 “Berlin criteria” proposed by the European Society of Intensive Care Medicine (ARDS Definition Task Force, 2012). Specifically, acute, progressive, bilateral pulmonary infiltrates visible on chest X-ray radiographs, alveolar capillary embedding pressure of 18 mmHg (1 mmHg = 0.133 kPa), and oxygenation index (OI = PaO_2_/FiO_2_) of 300 mmHg serve as indicators for ALI. However, the early stage of ALI in post-AAD patients might not exhibit discernible imaging changes, thereby limiting the utility of these criteria. As a result, there is a pressing demand for the identification of novel biomarkers that can predict the development and progression of ALI in AAD patients.

The complexity and heterogeneity of ALI pathophysiology necessitate the employment of high-throughput technologies, such as metabolomics, for pinpointing potential diagnostic and prognostic biomarkers for AAD with ALI. Metabolomics facilitates a comprehensive quantitative evaluation of small molecule metabolites (typically <1,500 Daltons) in cells, tissues, or organisms, which provides a direct functional readout of the physiological or pathological state of an organism (Nie et al., 2021). This study aims to leverage a metabolomics platform to identify diagnostic biomarkers for the early stage of AAD with ALI, address the perturbed pathways underlying disease progression, and illuminate the pathophysiological mechanisms of AAD with ALI.

## 2 Materials and methods

### 2.1 Patient selection

We conducted a retrospective study at the Department of Cardiac Surgery, Xiamen Cardiovascular Hospital of Xiamen University, involving 228 patients who underwent surgery between September 2019 and September 2022. These patients were diagnosed with acute lung injury (ALI), as confirmed by chest X-ray and PaO_2_/FiO_2_ assessments. The study was conducted in accordance with the approval of the Ethics Review Committee of the Xiamen Cardiovascular Hospital of Xiamen University. Written informed consent was obtained from all patients or their authorized representatives prior to the operation.

Initially, a total of 228 patients who had undergone preoperative computed tomographic angiography (CTA) were included in this retrospective study. However, patients with a history of CAD, coronary artery disease (CAD), acute left heart failure (ALHF), pneumonia, and diabetes and those with insufficient clinical data or blood samples were excluded from the study. After applying these exclusion criteria, 92 out of the initial 228 patients remained eligible for analysis. The patients were divided into two groups based on the results of chest X-ray and PaO_2_/FiO_2_ assessments (Supplementary Table S1). Furthermore, individuals with diabetes, cardiovascular diseases, or other conditions that could influence metabolic profiles were excluded from the study. Healthy individuals with a history or any signs of ALI based on chest X-ray or CT evaluation were excluded from the control group.

To conduct an unbiased search for plasma metabolites, the metabolomics analysis commenced. A total of 30 cases were randomly selected from 45 AAD patients diagnosed with ALI, and 30 cases were randomly selected from 47 AAD patients without ALI (non-ALI group). Additionally, an age- and gender-matched control group comprising 30 healthy individuals was randomly chosen. Detailed information regarding these subjects can be found in Supplementary Table S2. To ensure sample quality and facilitate result interpretation, plasma samples were collected from all participants 1 h postoperatively. Following collection, the samples were promptly separated and stored at −80°C.

### 2.2 Metabolite extraction

The metabolites were extracted by the addition of 400 μL of cold methanol/acetonitrile (1:1, v/v) to the sample, resulting in the removal of proteins. Thorough vortexing was performed to ensure proper mixing. For absolute quantification of metabolites, a stock solution of stable-isotope internal standard was added to the extraction solvent simultaneously (Supplementary Figure S1A). The mixture was transferred to a new centrifuge tube and centrifuged at 14,000 *g* for 20 min at 4°C to collect the supernatant. Subsequently, the supernatant was dried using a vacuum centrifuge. The dried samples were re-dissolved in 100 μL of acetonitrile/water (1:1, v/v) solvent and centrifuged at 14,000 *g* for 20 min at 4°C in preparation for LC-MS analysis.

### 2.3 LC-MS/MS analysis

The samples were analyzed using the ultra-high-performance liquid chromatography quadrupole-trap tandem mass spectrometry method (UHPLC-QTRAPMS). The UHPLC system used in this study was a 1290 Infinity System from Agilent Technologies (Santa Clara, CA, United States) coupled with a mass spectrometer QTRAP 6500+ MS from Sciex (Framingham, MA, United States).

For the separation of analytes, both HILIC (Waters UPLC BEH Amide column, 2.1 mm × 100 mm, 130 Å, 1.7 µm) and C18 columns (Waters UPLC BEH C18 column, 2.1 mm × 100 mm, 130 Å, 1.7 μm) were utilized. During HILIC separation, the column temperature was maintained at 35°C, and the injection volume was 2 μL. The mobile phase composition consisted of 90% H_2_O + 2 mM ammonium formate +10% acetonitrile (mobile phase A) and 0.4% formic acid in methanol (mobile phase B). A gradient elution was performed, starting with 85% B at 0–1 min, 80% B at 3–4 min, 70% B at 6 min, 50% B at 10–15.5 min, and finally reaching 85% B at 15.6–23 min. The flow rate was set at 300 μL/min. During RPLC separation, the column temperature was maintained at 40°C, and the injection volume was 2 μL. The mobile phase consisted of 5 mM ammonium acetate and 0.2% NH_3_·H_2_O (mobile phase A) and 99.5% acetonitrile and 0.5% NH_3_·H_2_O (mobile phase B). The gradient elution started with 5% B at 0 min, increased to 60% B at 5 min, reached 100% B at 11–13 min, and returned to 5% B at 13.1–16 min. The flow rate was set at 400 μL/min. Throughout the analysis process, the sample was maintained at 4°C.

The QTRAP 6500+ mass spectrometer was operated in positive and negative switch modes. For the positive mode, the source conditions are as follows: source temperature: 580°C; ion source gas 1 (GS1): 45; ion source gas 2 (GS2): 60; curtain gas (CUR): 35; IonSpray Voltage (IS): +4500 V. For the negative mode, the source conditions were as follows: source temperature: 580°C; ion source gas 1 (GS1): 45; ion source gas 2 (GS2): 60; curtain gas (CUR): 35; IonSpray Voltage (IS): −4500 V. Mass spectrometry quantitative data acquisition was performed using the multiple reaction monitoring (MRM) method. The MRM ion pairs used are listed in the attached file. To evaluate the stability and repeatability of the system, polled quality control (QC) samples were included in the sample queue. The relative standard deviations (%RSDs) in the pooled QC (n = 10) were calculated and presented in Supplementary Figure S1.

### 2.4 Metabolite identification and quantification

The raw data obtained from the mass spectrometry analysis were converted to the .mzML format using the ProteoWizard software. Subsequently, the XCMS program was employed for various data processing steps, such as peak alignment, retention time correction, and extraction of peak areas. During the peak picking step, the centWave algorithm was used with an m/z tolerance of 25 ppm. The peak width was set to a range between 10 and 60 data points, and a prefilter was applied in the range between 10 and 100 data points. For peak grouping, the bw parameter was set to 5, indicating the bandwidth for grouping similar peaks together. The mzwid parameter was set to 0.025, which represents the m/z width for grouping. The minfrac parameter was set to 0.5, indicating the minimum fraction of the samples in which a feature should be present to be considered for grouping. The metabolite structures were identified using exact mass number matching with a tolerance of less than 25 ppm. This matching was performed to compare the measured m/z values of the detected peaks with the expected mass values of known metabolites. Additionally, the secondary spectra matching was utilized for further identification of metabolite structures. This technique involves comparing the acquired spectra of the detected peaks with the reference spectra or databases to find matching patterns. All the aforementioned steps in data processing and metabolite identification aim to ensure accurate and reliable quantification of metabolites in the samples.

The metabolites were quantified in over half of the samples, indicating that some samples may have missing values for certain metabolites. To handle these missing values, the k-nearest neighbor (KNN) algorithm was applied for imputation. The KNN algorithm predicts missing values by considering the values of the nearest neighbors in the data set. The imputation process using the KNN algorithm was performed using the DMwR package in R version 3.5.3. After imputation, the expression levels of the metabolites were log-transformed. This transformation helps to address any skewness or heterogeneity in the data. Normalization and autoscaling were then applied to the log-transformed data. The quality control (QC) samples were processed together with the biological samples, monitoring any technical variations or instrument drift in the data analysis. To assess the reproducibility of the measurements, the coefficient of variation (CV) was calculated for each metabolite in the QC samples. Metabolites with a CV less than 30% were considered to have reproducible measurements. After performing these steps, the metabolomics data were robust and suitable for subsequent analysis and interpretation.

### 2.5 Statistical data analysis

Clinical statistical analyses were performed using the IBM SPSS software package (version 26.0; SPSS, Chicago, IL, United States). The clinical data were assessed for normal distribution using the Shapiro–Wilk test (*p* > 0.05). The normally distributed data were expressed as mean ± standard deviation, while the non-normally distributed or skewed data were presented as median [interquartile range]. The categorical data were reported as numbers (percentage). The Student’s t-test was used for normally distributed data, and the Mann–Whitney U test was employed for non-normally distributed or skewed data. The Pearson chi-square test or Fisher’s exact test was used for comparing numbers (percentage).

The significant variables (*p* < 0.05) identified through the univariate analysis (which included the Student’s t-test, Pearson chi-square test or Fisher’s exact test, and Mann–Whitney U test) were further analyzed using the multivariate analysis to estimate the odds ratios (ORs) for acute lung injury (ALI) and corresponding 95% confidence intervals (CIs). The selected variables (*p* < 0.05) from the multiple logistic regression analysis were included in the receiver operating characteristic (ROC) curve analysis.

The MetaboAnalyst 5.0 (https://genap.metaboanalyst.ca/MetaboAnalyst/) and R 4.2.3 (R Foundation for Statistical Computing, Austria) were utilized for metabolomic data analyses and visualizations after normalization and scaling. The multivariate data analysis, which included the Pareto-scaled principal component analysis (PCA), partial least squares discriminant analysis (PLS-DA), and sparse PLS discriminant analysis (sPLS-DA), was performed using the MetaboAnalyst. The robustness of the models was evaluated using tenfold cross-validation and response permutation testing. The variable importance in the projection (VIP) value of each variable in the PLS-DA model was calculated to indicate its contribution to the classification.

A two-tailed *t*-test was used to identify metabolites that differed between the groups, that is, ALI group vs. non-ALI group, ALI group vs. control group, and non-ALI group vs. control group. Notably, the *p*-values were corrected by the Benjamini–Hochberg algorithm. The differential metabolites were screened based on the criteria of an intersection of fold change >1.2 or <0.833 and *p*-value <0.05. The hierarchical cluster analysis was performed using the MetaboAnalyst 5.0 with Euclidean distances as the calculation of distances and Ward linkage as the clustering method.

Python (version 3.11.4) was used for important feature selection and classification model construction. Recursive feature elimination cross-validation (RFECV) was employed to calculate the important feature score using the random forest model in the sklearn.metrics package. A support vector machine (SVM) was applied to generate a mathematical model for discriminating between the ALI and non-ALI groups. The predictive performance of the model was assessed by estimating the area under the ROC curve (AUC) and confusion matrix, which were commonly used to evaluate the overall discriminant ability.

## 3 Results

### 3.1 Basic characteristics of patients

**FIGURE 1:**
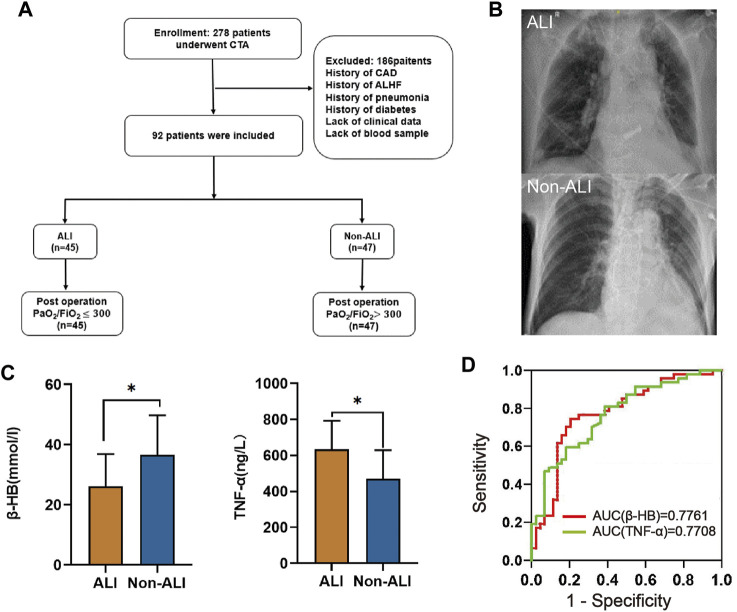
Study design and clinical cohort establishment. **(A)** In this retrospective study, we employed specific inclusion and exclusion criteria for the selection of AAD patients combined with acute lung injury (ALI). **(B)** Chest X-rays as a diagnostic measure for AAD patients with or without ALI were assessed. **(C)** The plasma levels of β-HB and TNF-α between the ALI and non-ALI groups were compared. **(D)** ROC analyses were conducted to evaluate the performance of β-HB and TNF-α as potential diagnostic biomarkers. Abbreviations: CTA: computed tomography angiography; CAD: coronary artery disease; ALHF: acute left heart failure.

Adherence to the inclusion and exclusion criteria resulted in the inclusion of 92 study participants (Figure 1A). The 92 study participants were divided into the ALI group and non-ALI group. The ALI group comprised patients exhibiting lung injury signs on chest X-rays, such as increased pulmonary edema and increased lung texture, along with a PaO_2_/FiO_2_ ratio of <300. On the other hand, the non-ALI group consisted of patients with a PaO_2_/FiO_2_ ratio of >300 (Figures 1A,B). The clinical characteristics of the patients are presented in Supplementary Table S1. The mean age was 53.7 years. A total of 64.1% of the patients had hypertension and 36% were smokers. Additionally, 12% of the patients had a history of alcohol consumption and 8% had false lumen thromboembolism. The breaking location was the ascending aorta in 49% of the cases. The median PaO_2_/FiO_2_ was 302.78 mmHg (IQR 93.7 years). Other median values included hs-CRP (3.21 mg/L), UA (404.8 mol/L), WBC (14.11*10^9^/L), PLT (171/L), D-Dimer (4.3 mg/L), HDL (1.05 mmol/L), glucose (7.83 mmol/L), BNP (133 pg/mL), LAC (1.3 mmol/L), ALT (19 U/L), AST (24.6 U/L), TG (1.05 mmol/L), CHOL (4.3 mmol/L), LDH (226.2 U/L), CKMB (14.4 ng/mL), CREA (82.1 umol/L), β-HB (29.6 mmol/L), IL-1β (48.1 ng/L), and IL-6 (30 pg/mL). The mean values for LDL, EF, IL-10, and TNF-α were 2.85 mmol/L, 67%, 758.2 pg/mL, and 549.3 ng/L, respectively (Supplementary Table S1). In order to explore more precise diagnostic biomarkers, we introduced metabolomic experiments, and the baseline of the 60 cases was aligned with that of the 92 cases (Supplementary Table S2).

### 3.2 Univariate analysis and ROC analysis

Through univariate and multivariate analyses to explore the relationship between the basic characteristics of the patients and ALI, we found that β-HB and TNF-α were independent risk factors for ALI (Table 1, Supplementary Table S1; Figure 1C). The ROC analysis showed that β-HB and TNF-α adequately discriminated the ALI patients with an AUC of 0.7761 (95% CI 0.68–0.88; *p*-value <0.0001) and 0.7708 (95% CI 0.68–0.87; *p*-value <0.0001) (Figure 1D).

**TABLE 1:** Multivariate analysis for acute lung injury in patients with acute aortic dissection.

Variables	B	SE	Wald χ^2^	*p*-value	OR	95% CI
DD (mg/L)	0.03	0.019	2.603	0.107	1.03	0.994–1.068
β-HB (mmol/L)	−0.098	0.032	9.59	0.002	0.906	0.852–0.965
IL-1β (ng/L)	0.015	0.016	0.924	0.336	1.015	0.984–1.047
IL-6 (pg/mL)	0.058	0.035	2.717	0.099	1.06	0.989–1.135
TNF-α (ng/L)	0.007	0.002	12.306	0	1.007	1.003–1.011
PaO_2_/FiO_2_ (mmHg)	−0.064	0.023	7.628	0.006	0.938	0.896–0.981
Breaking location^※^	0.579	0.489	1.401	0.236	1.784	0.684–4.561
ECC (min)	−0.001	0.004	0.11	0.741	0.999	0.992–1.006
AV (h)	0.004	0.004	0.827	0.363	1.004	0.995–1.013

Abbreviations: AV, assist ventilation; β-HB, β-hydroxybutyrate; B, regression coefficient; CI, confidence interval; DD, D-Dimer; ECC, extracorporeal circulation; FiO_2_, fraction of inspired O_2_; IL-1β, human interleukin-1 beta protein; IL-6, interleukin-6; OR, odds ratio; PaO_2_, partial pressure of oxygen; SE, standard error; TNF-α, tumor necrosis factor-α.

Breaking location^※^: location of aortic dissection vessel rupture.

### 3.3 Metabolite quantity and classification

**FIGURE 2:**
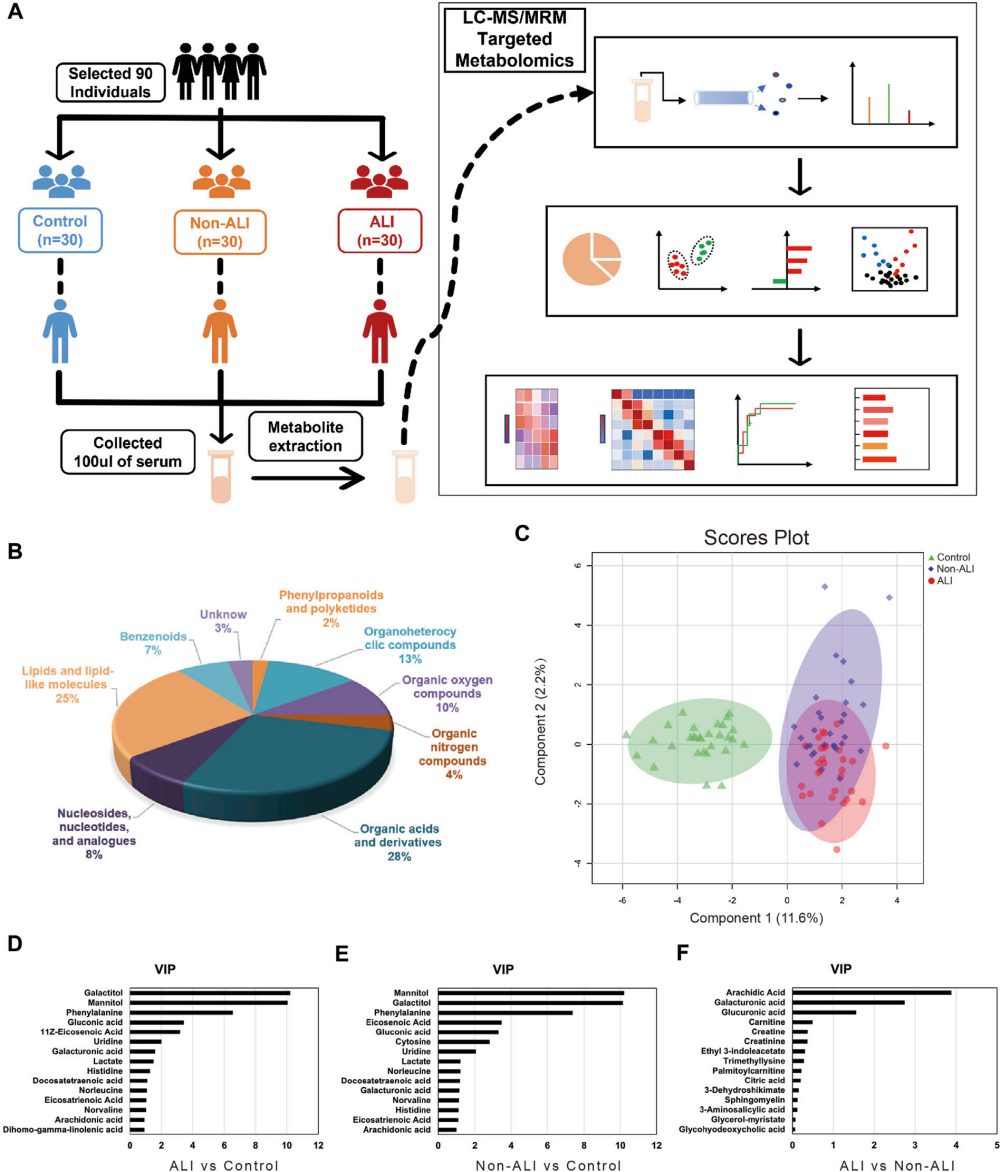
Workflow of this study and quantification of metabolites for groups between ALI patients, non-ALI, and healthy controls via the LC-MRM/MS approach. **(A)** Metabolites are accurately measured in clinical plasma samples using the targeted LC-MRM/MS approach, allowing researchers to identify a potential diagnostic panel for ALI. **(B)** A total of 418 metabolites from eight classes are identified by statistical analysis. **(C)** Based on the quantified metabolites, a PLS-DA score plot is used to distinguish between the control and ALI and non-ALI groups. **(D–F)** The top 15 metabolites ranked by VIP scores were presented from the ALI group vs Control group, Non-ALI group vs Control group, and ALI group vs Non-ALI group, respectively.

The workflow of this study and quantification of metabolites for groups between ALI patients, non-ALI, and healthy controls via the LC-MRM/MS approach is shown in Figure 2A. After data analysis, we obtained thousands of feature peaks, followed by the statistical analysis, and we identified a total of 417 metabolites from 8 classes (Figure 2B). Our results show that organic acids and derivatives contribute the highest percentage of metabolite composition at 28%, such as creatine, creatinine, ornithine, or sabinic acid. In addition, lipids and lipid-like molecules represented 16% of the total metabolites in this analysis, which included glycocholic acid and glycohyodeoxycholic acid. Additionally, organoheterocyclic compounds and organic oxygen compounds represented 13% and 10%, respectively (Supplementary Table S3).

### 3.4 Altered metabolic profiles among three groups

To identify metabolite variability between each group, PCA, PLS-DA, and sPLS-DA analyses were performed. The PCA score plots demonstrated a distinct separation between the ALI group vs. control group and non-ALI group vs. control group (Supplementary Figure S1A–C). To further investigate the differences in metabolites between the ALI group and non-ALI group, PLS-DA and sPLS-DA were conducted. The score plots of PLS-DA (Supplementary Figure S2D–F) and sPLS-DA (Figure 2C) showed clear distinctions between the three groups.

Based on the PLS-DA analysis, 15 metabolites with the highest VIP values were selected from the ALI group vs. control group (Figure 2D), non-ALI group vs. control group (Figure 2E), and ALI group vs. non-ALI group (Figure 2F). The top 10 metabolites with VIP values above 1 in both the ALI group vs. control group and non-ALI group vs. control group were considered discriminatory metabolites. Additionally, arachidic acid, galacturonic acid, and glucuronic acid with VIP values above 1.5 in the ALI group vs. non-ALI group were also identified as potential differentiating metabolites. The quality and validity of the PLS-DA (Supplementary Figures S3A–C) and sPLS-DA (Supplementary Figure 3G) models were evaluated through cross-validation, and the permutation test of PLS-DA also indicated the robustness of the models (Supplementary Figures S3D–F).

**FIGURE 3:**
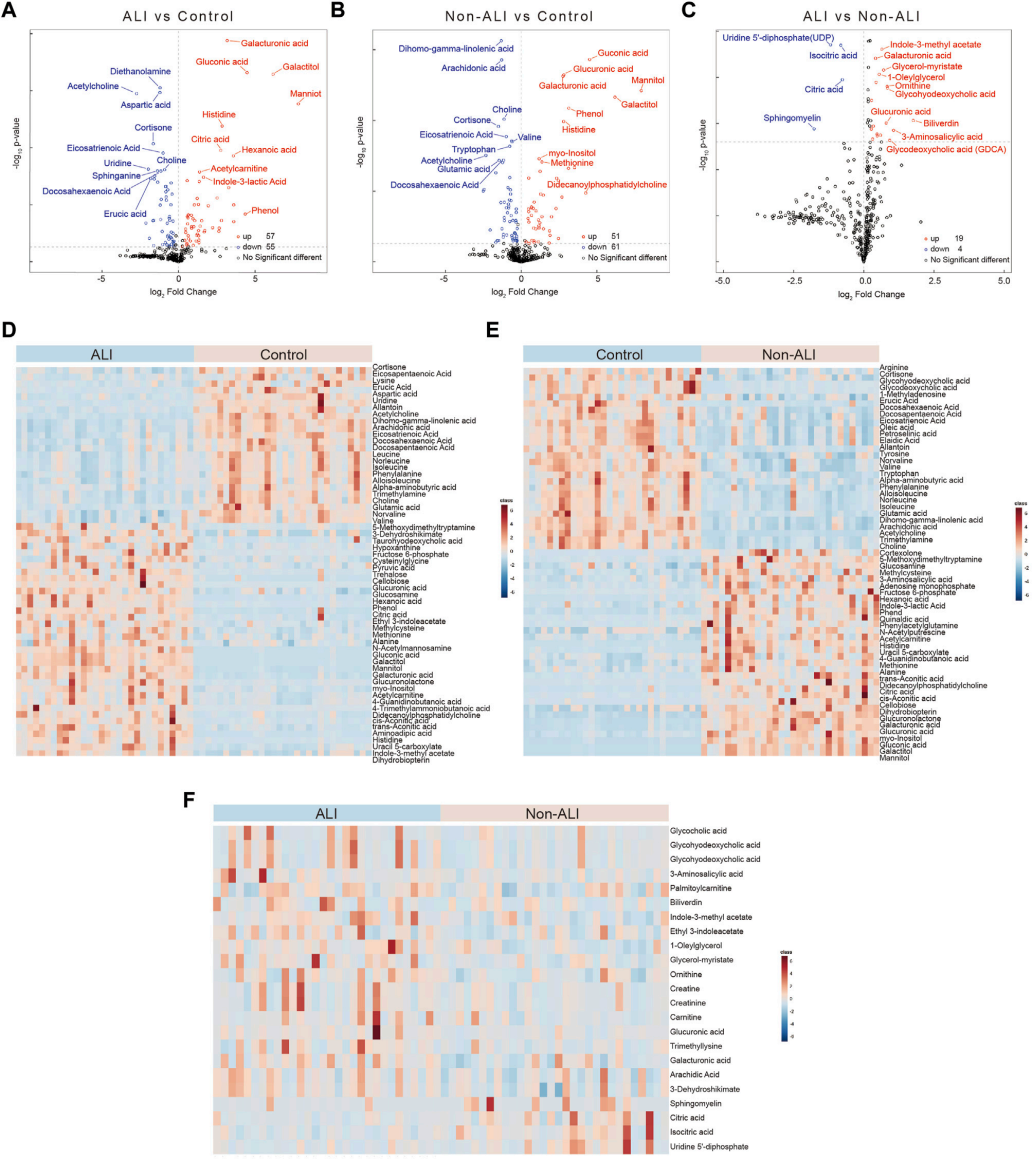
Volcano plots and heatmaps of the differential metabolites between three groups. **(A–C)** Volcano plots of the differential metabolites between ALI vs. control **(A)**, non-ALI vs. control **(B)**, and ALI vs. non-ALI **(C)**. **(D–F)** Significant differences for the variables among the three groups are depicted as heatmaps. Each row represents an individual metabolite, and each column represents an individual sample. Red represents the metabolites in high abundance, while blue represents the metabolites in low abundance.

Volcano plots were generated to visualize the overall changes in differential metabolites between the groups. These plots combined fold change (FC) with *p*-values, revealing a total of 57 upregulated and 55 downregulated metabolites in the ALI group vs. control group (Figure 3A), 51 upregulated and 61 downregulated metabolites in the non-ALI group vs. control group (Figure 3B), and 19 upregulated and 4 downregulated metabolites in the ALI group vs. non-ALI group (Figure 3C). The hierarchical clustering analysis was performed to provide a visual representation of the metabolite expression variation. The heatmaps of the ALI group vs. control group (Figure 3D) and non-ALI group vs. control group (Figure 3E) both clearly demonstrated the differences in the metabolite profiles. However, the heatmap of the ALI group vs. non-ALI group showed less distinct clustering, suggesting a less pronounced differentiation than in the two other groups (Figure 3F).

Furthermore, based on fold change (FC > 1.2 or <0.833) and *p-*value (*p* < 0.05) criteria, the top 20 metabolites with the most differential expressions were selected in each group (Supplementary Figures S4A–C). In detail, 36 metabolites were more than twofold upregulated and 22 metabolites were more than twofold downregulated in the ALI group than in the control group (Supplementary Table S4). A total of 32 metabolites were more than twofold upregulated and 23 metabolites were more than twofold downregulated in the non-ALI group than in the control group (Supplementary Table S5). Likewise, when compared to the non-ALI group, biliverdin was more than threefold upregulated in the ALI patients, and 3-aminosalicylic acid was more than twofold upregulated, while sphingomyelin and uridine 5′-diphosphate (UDP) were more than twofold downregulated (Supplementary Table S6). Collectively, the aforementioned analyses and visualizations revealed significant differences in metabolic profiles between the ALI group, non-ALI group, and control group. Several discriminatory metabolites were identified, providing insights into the metabolic alterations associated with ALI.

### 3.5 Potential biomarkers for ALI diagnosis

**FIGURE 4:**
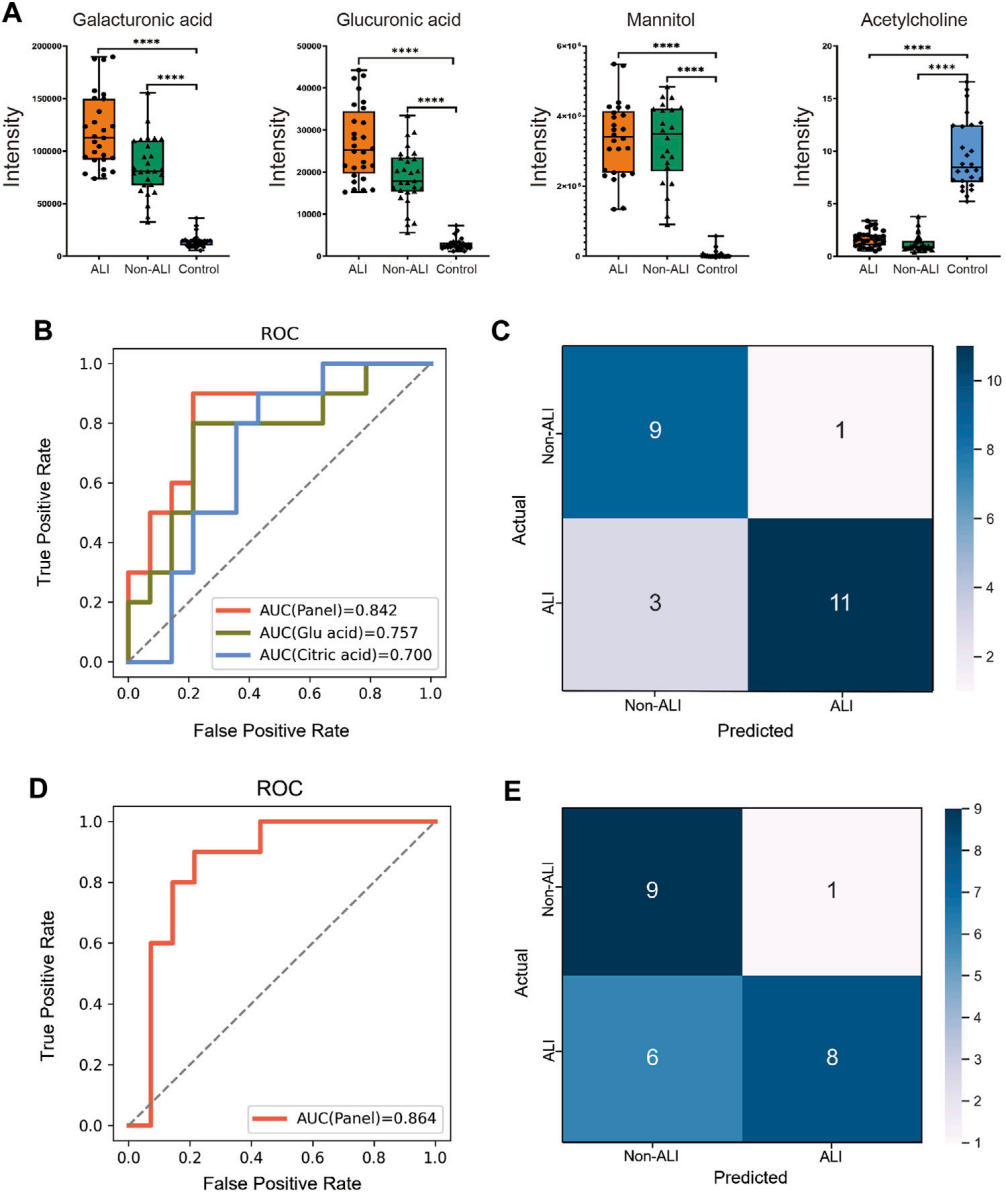
Diagnostic panel for differentiating between the ALI and non-ALI groups. **(A)** Bar charts illustrate the intensities of four metabolites, namely, galacturonic acid, glucuronic acid, mannitol, and acetylglycine, across the three groups. **(B)** ROC curve analysis is performed for citric acid, glucuronic acid, and the combination of these two metabolites is presented as a panel. **(C)** SVM method is employed to generate a confusion matrix displaying the classification results. **(D)** Incorporating β-HB and TNF-α into the panel of two metabolites and re-evaluating the model, a slightly higher AUC of 0.864 is observed. **(E)** This incorporated model’s accuracy (0.7083), precision (0.5816), and sensitivity (0.571) are all decreased when compared with the model that only contains two metabolites—citric acid and glucuronic acid.

The box plots demonstrated the intensities of the four selected metabolites (galacturonic acid, glucuronic acid, mannitol, and acetylcholine) among the three groups, as evidenced by *p*-values <0.001 for all comparisons (Figure 4A). Additionally, all four metabolites exhibited AUC values exceeding 0.95 in the ROC analyses conducted for both groups (Supplementary Figure S4). Consequently, all four metabolites hold the potential as biomarkers for distinguishing between the ALI group vs. control group and non-ALI group vs. control group.

To focus on biomarker discovery, specifically in the ALI group vs. non-ALI group, a combination of criteria was applied. The intersection of the top 20 metabolites based on fold change (FC > 1.2 or <0.833) with *p*-value <0.05 (Supplementary Figure S5A–C) and the top 15 metabolites based on VIP in PLS-DA (Figure 2F) was chosen for the RFECV analysis. This analysis identified 12 metabolites, and citric acid obtained the highest feature score (Supplementary Figure S5D).

The best biomarker panel was selected through cross-validation of random combinations of these 12 metabolites. The combination of citric acid and glucuronic acid achieved the highest score of 0.80158 (Supplementary Figure S5E). The 60 samples were randomly divided into training and test groups (6:4 ratio), and an SVM classification model was used to verify the classification performance of the biomarker panel. The ROC curve demonstrated that combining citric acid and glucuronic acid as biomarkers (AUC = 0.842) resulted in a higher accuracy than when using citric acid (AUC = 0.700) or glucuronic acid (AUC = 0.757) individually (Figure 4B). The confusion matrix of the SVM model showed that out of 24 individuals, 4 were misclassified (Figure 4C). In our cohort, the accuracy, precision, F1-score, sensitivity, and specificity of the model were calculated as 0.8333, 0.7166, 0.8181, 0.786, and 0.900, respectively.

Furthermore, we incorporated β-Hb and TNF-α into the panel of metabolites and re-evaluated the model, resulting in a slightly higher AUC of 0.864 (Figure 4D). However, upon examining the confusion matrix, we observed that the performance was inferior when compared to the panel of the two initial metabolites (citric acid and glucuronic acid). Additionally, the model’s accuracy (0.7083), precision (0.5816), and sensitivity (0.571) decreased (Figure 4E). Based on these findings, we concluded that combining citric acid and glucuronic acid as a biomarker panel improved the classification performance for distinguishing between the ALI group and non-ALI group.

### 3.6 Altered metabolic pathways

**FIGURE 5:**
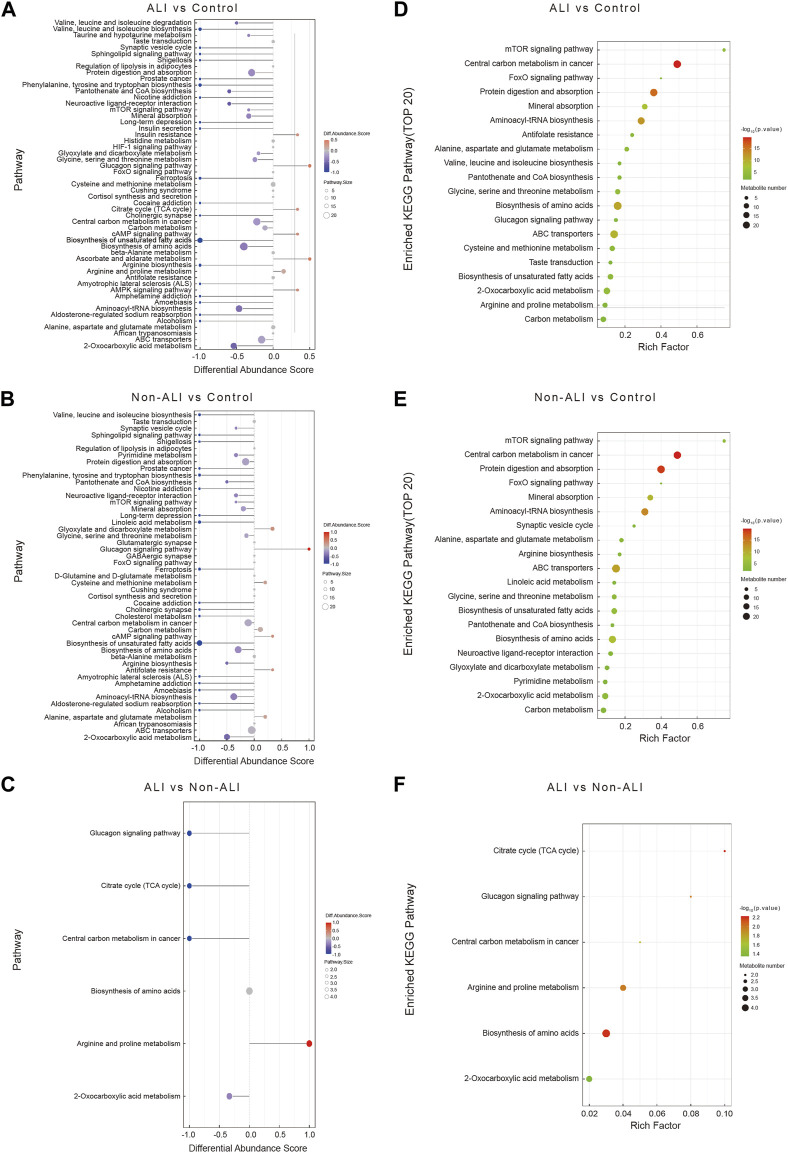
A comprehensive analysis to assess the metabolic pathways in the three groups. **(A–C)** The differential abundance score map (DA Score) illustrates the differential metabolic pathways between ALI group vs. control group **(A)**, non-ALI group vs. control group **(B)**, and ALI group vs. non-ALI group **(C)**. In the map, the red color indicates an upregulated trend in the expression of all identified metabolites within the pathway, while blue represents a downregulated trend. The length of the line corresponds to the absolute value of the DA score, and the size of the dots at the end of the line represents the number of metabolites in the pathway. **(D–F)** Enrichment analysis is further used to investigate the metabolic pathway between the ALI group vs. control group **(D)**, the non-ALI group vs. control group **(E)**, and the ALI group vs. non-ALI group **(F)**. Intensity of the red color indicates the significance of enrichment, and the rich factor denotes the proportion of differential metabolites in the pathway when compared to the total number of metabolites annotated in that pathway.

The DA score analysis was performed using the MetaboAnalyst to assess the differential metabolic pathways between the ALI group vs. control group (Figure 5A), the non-ALI group vs. control group (Figure 5B), and the ALI group vs. non-ALI group (Figure 5C). These DA score maps provided insights into alterations in the pathways and pathway metabolites across the three groups.

To further identify the significantly affected metabolic and signal transduction pathways, the enrichment analysis was conducted by using the R package, and the significance levels of metabolite enrichment for each pathway were calculated. This analysis resulted in an enrichment pathway map for each group (Figures 5D–F). Notably, several biological processes, such as the tricarboxylic acid cycle (TCA), biosynthesis of amino acids pathways, glucagon signaling pathway, arginine and proline metabolism, and 2-oxocarboxylic acid metabolism, were significantly altered in all three groups.

**FIGURE 6:**
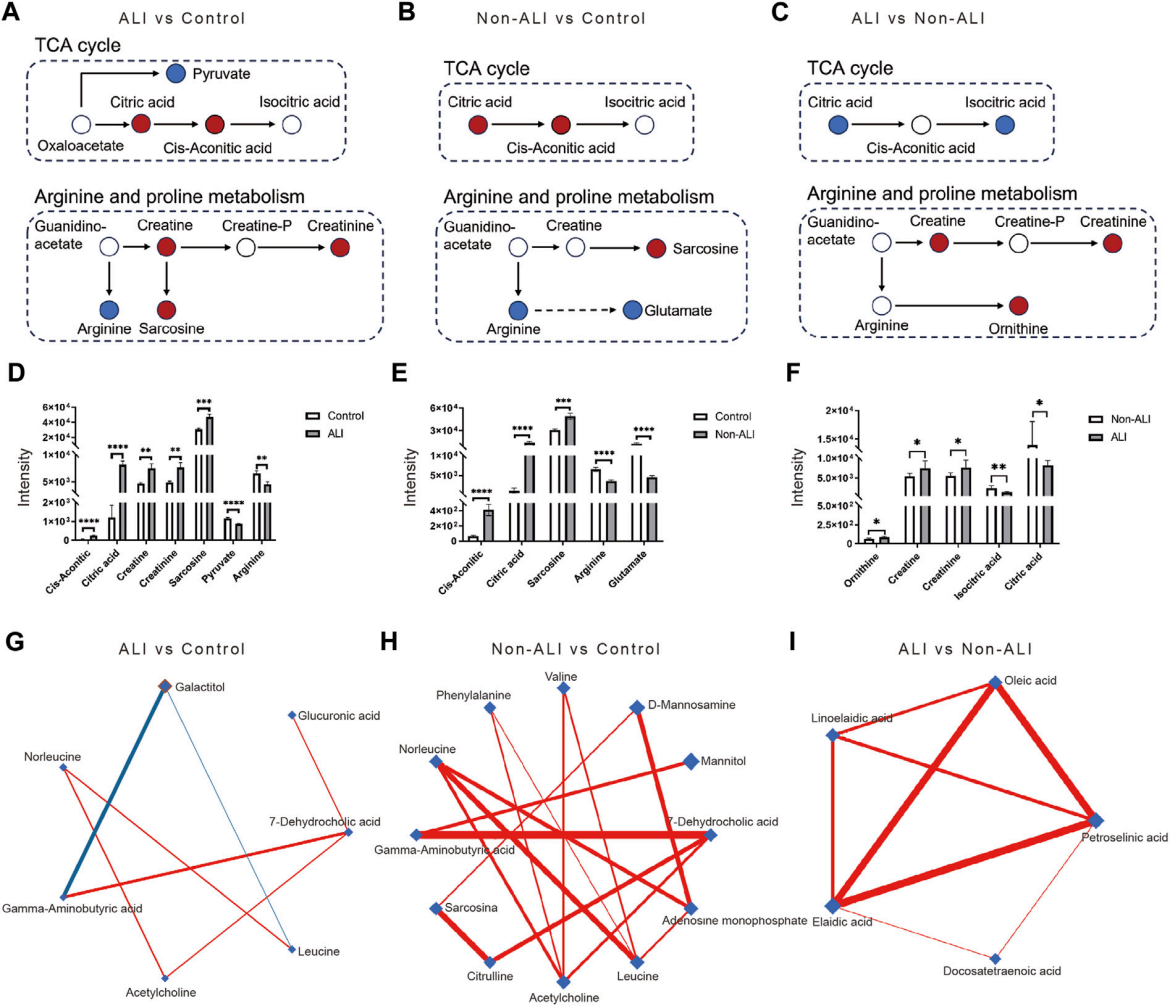
Key metabolic pathways involved in AAD patients with or without ALI, along with the differential metabolites identified in these pathways. **(A–C)** In the comparisons of ALI group vs. control group **(A)**, the non-ALI group vs. control group **(B)**, and the ALI group vs. non-ALI group **(C)**, upregulated metabolites are represented by red circles, while downregulated metabolites were denoted by blue circles in the TCA cycle as well as the arginine and proline metabolism pathways. **(D–F)** Bar charts are generated to visualize the intensities of differential metabolites between the ALI vs. control **(D)**, non-ALI vs. control **(E)**, and ALI vs. non-ALI **(F)**. **(G–I)** De-Biased Sparse Partial Correlation (DSPC) network analysis is performed for the differential metabolites in the ALI group vs. control group, non-ALI group vs. control group, and ALI group vs. non-ALI group. Metabolites are represented as nodes, and the edges depict the partial correlation of DSPC between two metabolites after conditioning on all other metabolites. The width of the edges represent the strength of the partial correlation coefficient.

The key metabolic pathways, such as the TCA cycle and arginine and proline metabolism, along with the differential metabolites identified in these pathways, were presented for the ALI group vs. control group (Figure 6A), non-ALI group vs. control group (Figure 6B), and ALI group vs. non-ALI group (Figure 6C). The intensities of metabolites, such as creatine, creatinine, and others, exhibited significant alterations among the three groups (Figures 6D–F).

Additionally, the De-Biased Sparse Partial Correlation (DSPC) network analysis was conducted using MetaboAnalyst 5.0 to explore the correlation between the metabolites. In the ALI group vs. control group, the metabolites 7-dehydrocholic acid, galactitol, acetylcholine, glucuronic acid, and gamma-aminobutyric acid showed close correlations, with galactitol being negatively correlated with gamma-aminobutyric acid (Figure 6G). Similarly, in the non-ALI group vs. control group, 7-dehydrocholic acid, acetylcholine, and gamma-aminobutyric acid exhibited dense correlations (Figure 6H). However, in the ALI group vs. Non-ALI group, the correlations between elaidic acid, oleic acid, linoelaidic acid, and petroselinic acid were worth exploring further (Figure 6I).

Overall, these analyses provided comprehensive insights into the differential metabolic pathways, key metabolites, and correlations between metabolites in the ALI group, non-ALI group, and control group.

## 4 Discussion

The occurrence of ALI severely affects the prognosis of patients with AAD (Zhang et al., 2019). AAD is a subset of thoracic aortic dissection (TAD) that specifically refers to dissections occurring in the ascending aorta, while TAD is a broader term that encompasses dissections in different sections of the thoracic aorta. TAD is a serious medical condition that involves a tear in the inner layer of the aorta, a major blood vessel responsible for delivering oxygenated blood from the heart to the entire body. This tear creates a false channel within the aortic wall, allowing blood to flow between the layers of the vessel wall. As a result, TAD poses a severe risk to life and necessitates urgent medical attention and intervention to address the condition effectively.

Metabolomics has witnessed rapid advancements and offers a comprehensive and unbiased methodology for identifying metabolic alterations associated with AAD and TAD. Several metabolite alterations, such as upregulated plasma succinate and trimethylamine N-oxide (TMAO) and decreased LPCs and sphingolipids were observed in AAD patients and regarded as potential biomarkers (Zhou et al., 2019; Cui et al., 2021; Zeng et al., 2020; Yang et al., 2020). However, these metabolomic findings are rarely correlated with prognosis in patients with AAD.

The role of inflammation in the progression of AAD is indeed widely recognized (Sugano et al., 2005; Kurabayashi et al., 2010; Chen et al., 2016). This study found that elevated preoperative levels of β-HB and TNF-α were associated with AAD combined with ALI. β-HB, a signaling metabolite that inhibits the activation of the NLRP3 inflammasome (Wei et al., 2022), has the potential to serve as an early biomarker for ALI. TNF-α, a well-known inflammatory indicator, has also been implicated in ALI in previous studies (Kurabayashi et al., 2010).

It is important to note that while β-HB and TNF-α show promise as potential biomarkers, further research using high-throughput technologies is required to identify more accurate biomarkers for the identification of AAD patients combined with ALI. Metabolomic analysis can provide valuable insights into the metabolic alterations associated with this condition. Our study identified relevant metabolic alterations in several pathways, such as in the TCA cycle, amino acid biosynthesis, protein digestion and absorption, ABC transporter, and glucagon signaling pathway. These pathways are known to play important roles in cellular metabolism, inflammation, and tissue damage.

The TCA cycle constitutes the center of cellular metabolism and supports the basic functions of cellular bioenergetics (Williams and O’Neill, 2018). The abundance of different TCA cycle metabolites has emerged as a crucial determinant of cellular function and fate in diverse contexts (Martínez-Reyes and Chandel, 2020). The TCA cycle has also been recognized as an important component of glycolysis, and perturbations in the TCA cycle metabolites can have profound effects (Fernie et al., 2004). The accumulation of metabolites from the TCA cycle, particularly succinate, can contribute to a vascular inflammatory response by activating inflammatory signaling pathways, inducing oxidative stress, and modulating immune cell functions (Williams and O’Neill, 2018; Cui et al., 2021). Recent evidence demonstrate that enhancing glycolysis in alveolar epithelial cells during lung tissue injury can help maintain cellular energy balance, reduce oxidative stress, regulate inflammation, and support cell repair processes (Ning et al., 2023). Our findings indicate a downregulation of TCA cycle metabolites and disrupted degradation of citric acid, which may contribute to lung function damage in AAD combined with ALI. These metabolic alterations reflect the dysregulation of energy metabolism and cellular function in the lung tissue of these patients.

The role of amino acids in cellular processes, such as protein synthesis, energy production, and cell repair, is well established (Holeček, 2018; Yang et al., 2020). Alterations in plasma amino acid levels have been associated with various diseases, and specific amino acids have been identified as biomarkers for disease susceptibility, severity, and recovery. For example, arginine and proline have been recognized as critical biomarkers in acute respiratory distress syndrome (ARDS) (Viswan et al., 2017). The upregulation of L-proline transport can further provide the necessary precursors required for vascular SMC collagen deposition and growth, promoting arterial remodeling at the site of vascular injury (Ensenat et al., 2001; Reyna, 2004). L-lysine has shown protective effects against acute lung injury (ALI) in animal models (Zhang et al., 2019). The dysregulation of amino acids may contribute to the development and progression of ALI in the context of AAD.

Pulmonary surfactant, composed of lipids and proteins, is essential for maintaining lung function and preventing atelectasis (Wang et al., 2021). The loss of pulmonary surfactant leads to decreased pulmonary compliance and edema in ALI (Cattel et al., 2021). The glucagon signaling pathway and ABC transporter pathway are implicated in the regulation of pulmonary surfactant. The glucagon-like peptide-1 (GLP-1) analog has been shown to increase pulmonary surfactant expression (Sato et al., 2020), suggesting the involvement of the glucagon signaling pathway in ALI. Additionally, ABC transporters, highly expressed in the lung, are associated with surfactant deficiency (Van Der Deen et al., 2005). ABC transporters have been confirmed to be involved in cholesterol homeostasis, blood pressure regulation, endothelial function, vascular inflammation, and platelet production and aggregation (Schumacher and Benndorf, 2017). Consistent with our results, the ABC transporter pathway may play crucial roles in the early stages of ALI development in the context of AAD.

Hyaluronic acid (HA) is involved in tissue repair and wound healing processes (Toole, 2004; Papakonstantinou et al., 2012). The circulating and alveolar levels of HA, composed of D-glucuronide and N-acetylglucosamine, have been found to correlate with the severity of lung injury (Viswan et al., 2017; Sato et al., 2020). Glucuronic acid, a component of HA, may have an important role in regulating ALI in the context of AAD.

It is important to acknowledge the limitations of our study. The sample size was relatively small, and further validation in a larger population is necessary. Being a retrospective study, potential misclassification bias cannot be completely ruled out. Additionally, the effects of surgery itself on lung tissue should be considered, and including postoperative ALI cases in future studies would provide a more comprehensive understanding of the metabolic changes associated with AAD combined with ALI.

## 5 Conclusion

In conclusion, our study provides valuable insights into the metabolic alterations in patients with AAD combined with ALI. We identified significant changes in metabolic pathways, such as the TCA cycle, amino acid biosynthesis, protein digestion and absorption, ABC transporter, and the glucagon signaling pathway. Specifically, we observed significant variations in the concentrations of β-HB, TNF-α, citric acid, and glucuronic acid in AAD patients accompanied by ALI. These findings contribute to the early identification and evaluation of ALI in AAD patients.

However, further research is required to validate these identified biomarkers and understand the precise mechanisms underlying the pathophysiology of ALI in AAD patients. Future studies should involve larger patient cohorts and consider other factors that may influence metabolic alterations in order to enhance the clinical relevance and applicability of these findings. Additionally, functional studies and mechanistic investigations are warranted to gain a deeper understanding of the metabolic pathways involved in AAD patients with ALI.

## Data Availability

The original contributions presented in the study are included in the article/Supplementary Material; further inquiries can be directed to the corresponding authors.
