# Supplementary material for: Metabolomic characterization benefits the identification of acute lung injury in patients with type A acute aortic dissection
Source: Front Mol Biosci. 2023 Aug 3;10:1222133. doi: 10.3389/fmolb.2023.1222133 (PMC10434778; doi:10.3389/fmolb.2023.1222133)
Supplement: Supplementary file 2 [file Table1.DOCX]

**Supplementary materials**

**Metabolomic characterization benefits the identification of acute lung injury in patients with type A acute aortic dissection**

Linglin Fan^#^, Ke Meng^#^, Fanqi Meng, Yuan Wu*, Ling Lin*

**Supplementary Figure 1**


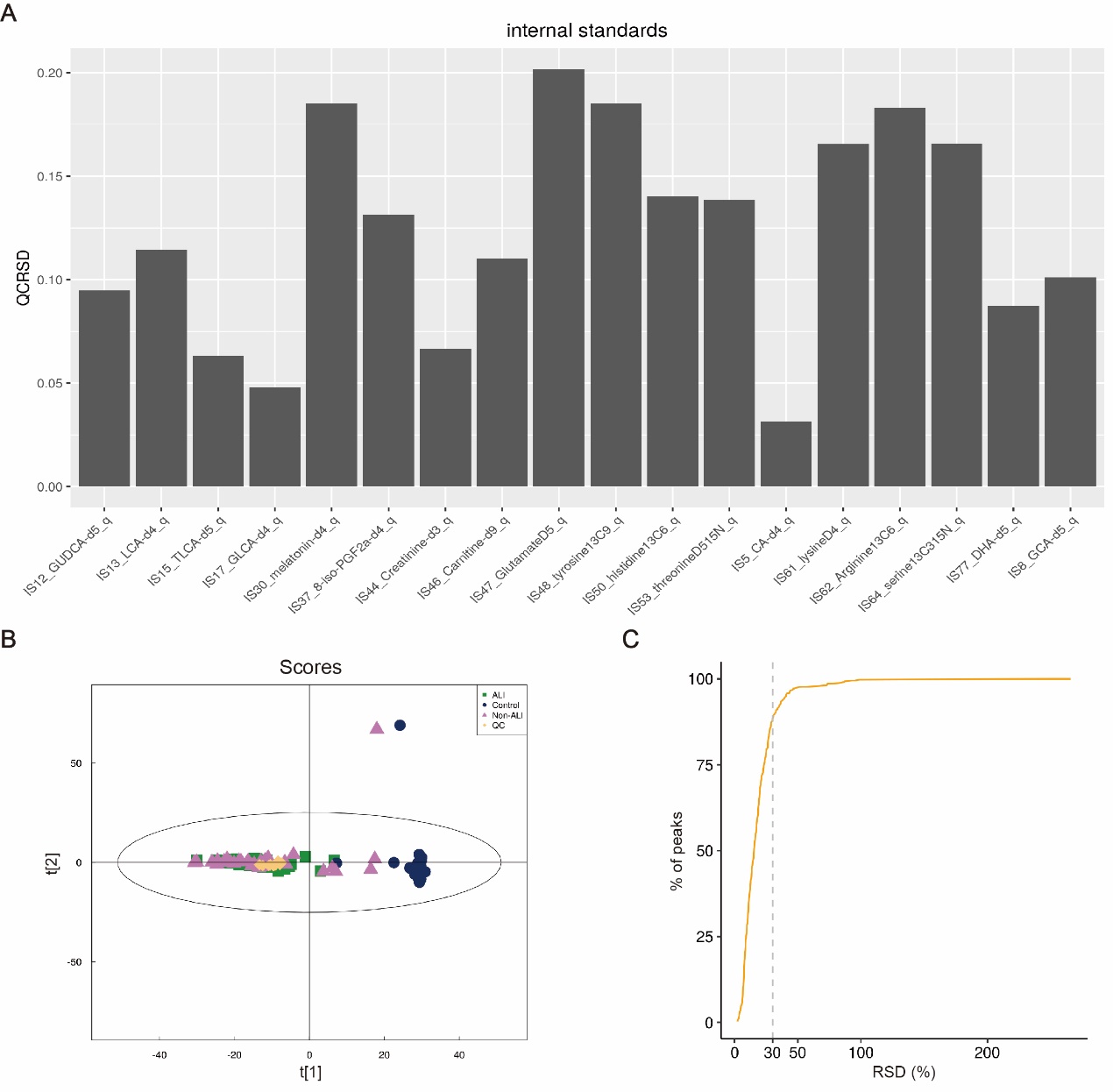


**Supplementary Figure 1** Isotope internal standard method and QC quality control. (A) Stability evaluation plots of isotopic internal standard responses in QC samples. The RSD value (standard deviation) distribution was calculated from the response of the internal standard in the QC samples, with the smaller the RSD (RSD ≤ 20%), the more stable the system is. (B) PCA analysis of the overall sample. The QC samples were closely clustered together, indicated good reproducibility of the experiment. (C) RSD distribution of QC samples. The horizontal coordinate represents the range of RSD and the vertical coordinate reflects the peak number of RSD in a certain range, the higher the percentage of RSD less than 30% (≥ 80%), the system more stable.

**Supplementary Figure 2**

**
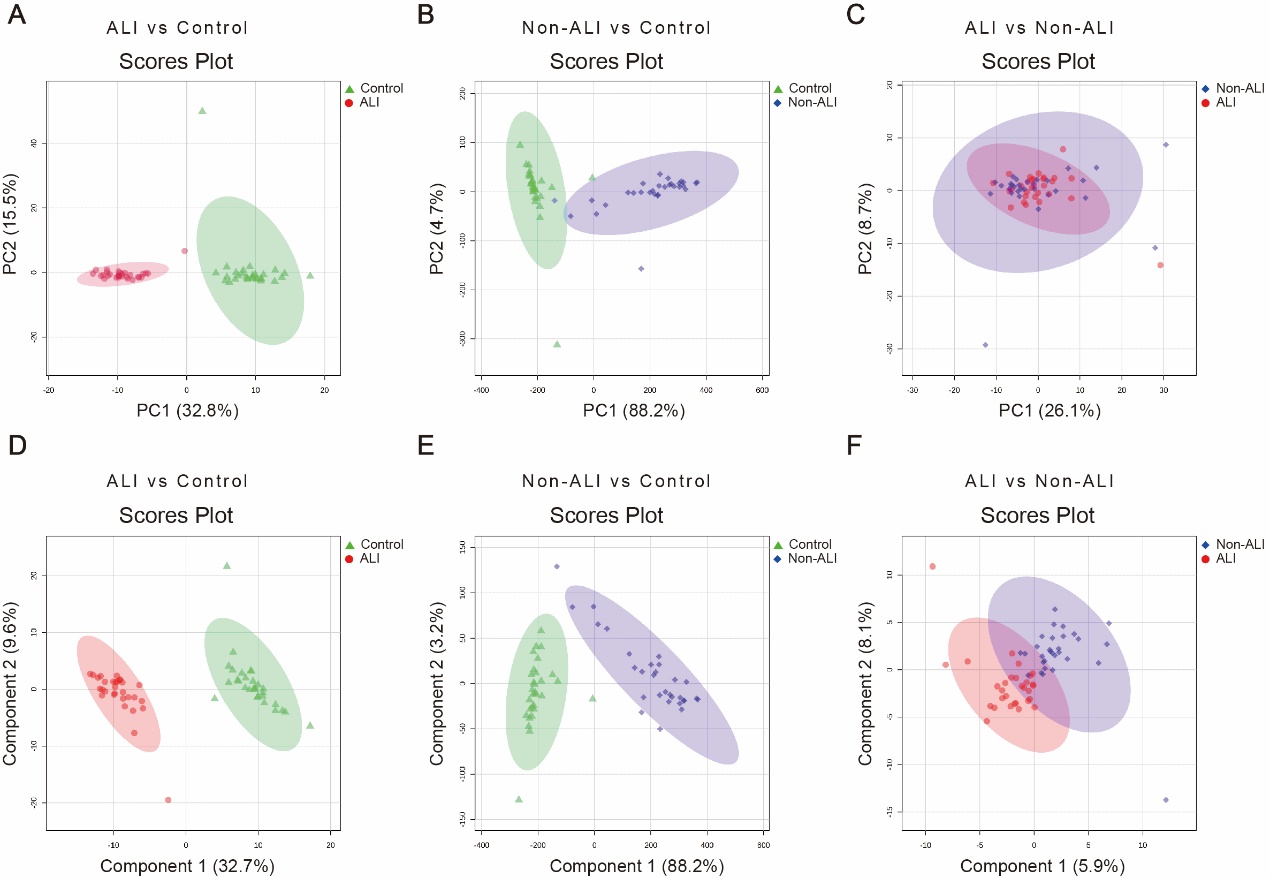
**

**Supplementary Figure 2.** PCA and PLS-DA scores plots based on three groups. (A-C) PCA scores plot between Control group vs ALI group, Control group vs Non-ALI group, ALI group vs Non-ALI group. (D-F) PLS-DA scores plot between Control group vs ALI group, Control group vs Non-ALI group, ALI group vs Non-ALI group.

**Supplementary Figure 3**

**
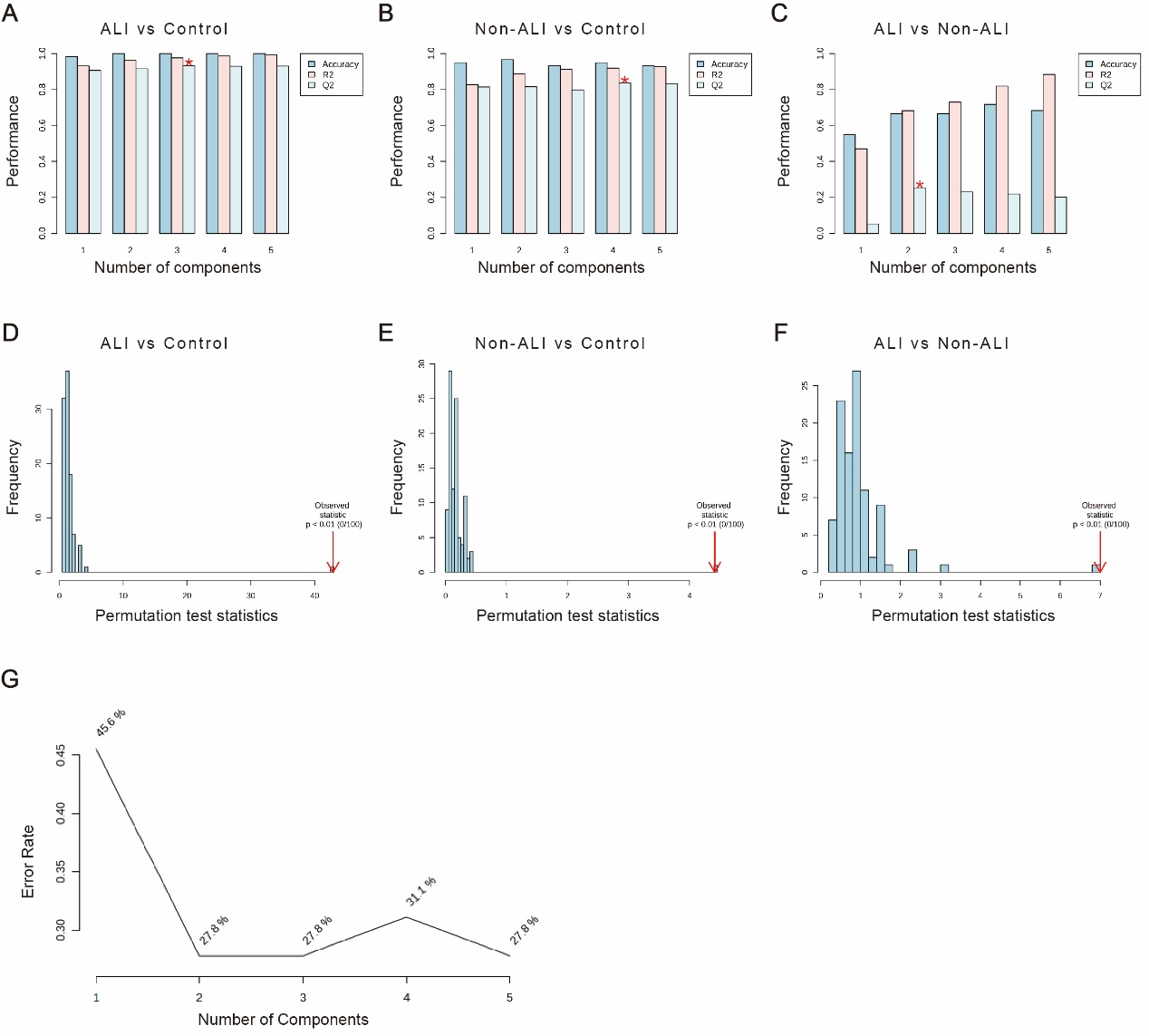
**

**Supplementary Figure 3.** Cross-validation and permutation tests to examine the quality of PLS-DA and sPLS-DA models. (A-C) Accuracy, R^2^, Q^2^ of the PLS-DA models under 10-fold cross-validation between Control group vs ALI group (A), Control group vs Non-ALI group (B), ALI group vs Non-ALI group (C). (D-F) Permutation test statistics of the PLS-DA models between Control group vs ALI group (D), Control group vs Non-ALI group (E), ALI group vs Non-ALI group (F). (G) Ten-fold cross-validation of the sPLS-DA model.

**Supplementary Figure 4**

**
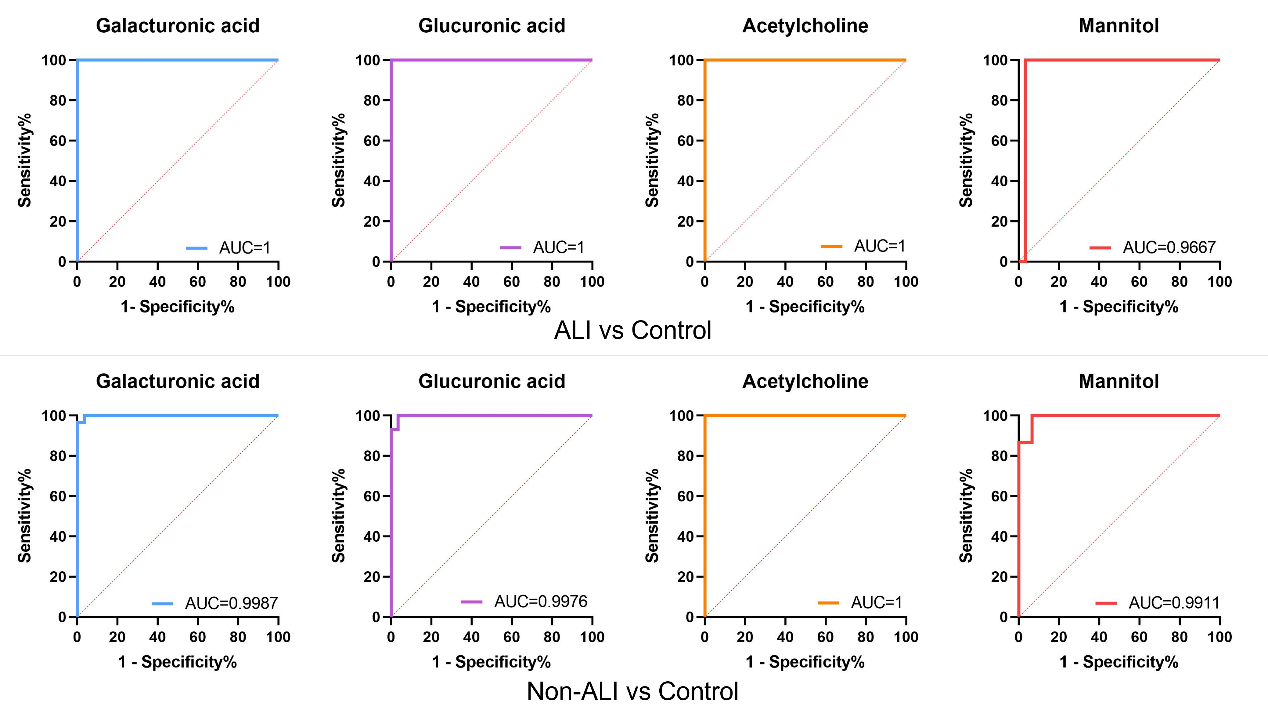
**

**Supplementary Figure 4.** All four metabolites (Galacturonic acid, Glucuronic acid, Mannitol, Acetylcholine) exhibited AUC values exceeding 0.95 in the ROC analyses conducted for both groups, distinguishing between the ALI group vs Control group and Non-ALI group vs Control group.

**Supplementary Figure 5**

**
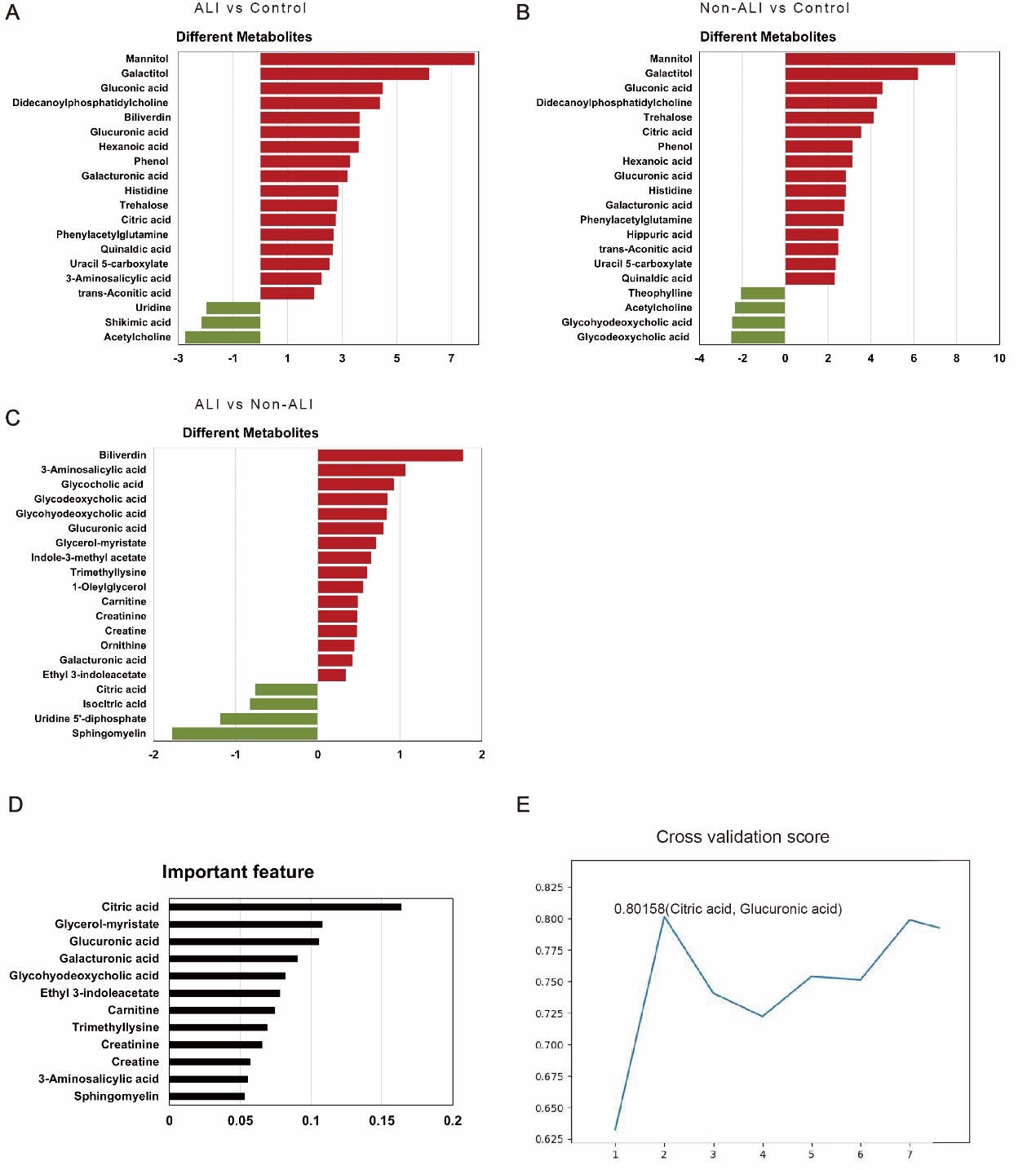
**

**Supplementary Figure 5.** Fold change of the differential metabolites and important feature selection. (A-C) The top 20 differential metabolites between the Control group vs ALI group, Control group vs Non-ALI group, ALI group vs Non-ALI group. (D) The targeted metabolites for biomarker selection with important feature scores by RFECV. (E) Cross validation obtained by randomly combining 20 metabolites from RFECV.
