# Supplementary material for: Metabolomic characterization benefits the identification of acute lung injury in patients with type A acute aortic dissection
Source: Front Mol Biosci. 2023 Aug 3;10:1222133. doi: 10.3389/fmolb.2023.1222133 (PMC10434778; doi:10.3389/fmolb.2023.1222133)
Supplement: Supplementary file 3 [file Table2.DOCX]

**Supplementary Table S1.** Baseline characteristics of the patients (N = 92). Univariate analysis in ALI.

| Variables | | Total (N=92) | ALI (N=45) | Non-ALI (N=47) | T | *P*-value |
| --- | --- | --- | --- | --- | --- | --- |
| Gender (male) | | 72(78.3%) | 35(77.8%) | 37(78.7%) | 0.012 | 0.912 |
| Age (years) | | 53.7± 11.8 | 53.2±10.61 | 54.3±12.36 | 0.495 | 0.622 |
| HBP | | 59(64.1%) | 31(68.89%） | 33(70.21%） | 0.022 | 0.833 |
| DM | | 0.000 | 0.000 | 0.000 | 0.000 | 0.000 |
| Smoking | | 49(53.3%) | 22(48.89%) | 27(57.45%) | 0.653 | 0.419 |
| Drinking | | 12(12%) | 8(17.78%) | 3(6.38%) | 2.971 | 0.243 |
| Break location^※^ | |  |  |  |  |  |
| Asc | | 49(53.3%) | 18(40%) | 31(66%) | 4.783 | 0.029 |
| Non-Asc | | 40(43.5%) | 24(53.3%) | 16(34%) |  |  |
| FLTE | | 44(47.8%) | 21(46.7%) | 23(48.9%) | 0.050 | 0.823 |
| PaO_2_ (mmHg) | | 97.5(75.2~134) | 75.5(62.1~89.3） | 50.3(30.4~121.75） | -2.728 | 0.006 |
| PaO_2_/FiO_2_ (mmHg) | | 302.78(223.75~411.11） | 223.75(185.16~254.72) | 85.8(55.6~199.3) | -4.083 | <0.001 |
| hs-CRP (mg/L) | | 3.21(2~7.41） | 4.46(2.22~12.48) | 4.17(2.05~16.2) | -0.214 | 0.831 |
| UA (mol/L) | | 404.8(294.2~507.5） | 422.8 (338.95~535.55) | 392.95 (275.6~497.85) | -1.445 | 0.149 |
| WBC (/L) | | 14.11(12.03~16.39） | 14.27(12.215~16.375) | 13.325(11.273~16.74) | 0.991 | 0.365 |
| PLT (/L) | | 171(133~206) | 167(128~206.5) | 174(149.5~204.25) | -0.595 | 0.552 |
| DD (mg/L) | | 4.325(2.633~25.36) | 15.33 (3.43~28.48) | 3.55 (1.615~28.19) | -2.240 | 0.025 |
| LDL (mmol/L) | | 2.846±0.663 | 2.88±0.66 | 2.81±0.67 | -0.054 | 0.616 |
| HDL (mmol/L) | | 1.05(0.96~1.31) | 1.04(0.93 1.32) | 1.08(0.99 1.31) | -0.759 | 0.448 |
| GLU (mmol/L) | | 7.83(6.82 9.16) | 7.71(6.83~8.68) | 7.92(6.82~9.26) | -0.254 | 0.799 |
| BNP (pg/mL) | | 133 (81.8~266) | 151.5(84.08~277) | 125(76.4~247) | -0.814 | 0.416 |
| LAC (mmol/L) | | 1.3(0.9~2.4) | 1.4(1~2.75) | 1.2(0.8~2.1) | -1.161 | 0.245 |
| ALT (U/L) | | 18.95(13.9~28.53) | 20.7 (16.5~27) | 16.9(13.1~31.4) | -0.533 | 0.594 |
| AST (U/L) | | 24.6(20.6~31.1) | 24.2(20.7~31) | 26.1(20.3~31.1) | -0.647 | 0.517 |
| TG (mmol/L) | | 1.05(0.81~1.66) | 1.02(0.83~1.82) | 1.05(0.77~1.47) | -0.369 | 0.712 |
| CHOL (mmol/L) | | 4.34(3.87~5.05) | 4.35(3.86~5.1) | 4.31(3.87~5.40) | -0.544 | 0.586 |
| LDH (U/L) | | 226.2 (201.4~267.8) | 232.25 (201.8~282) | 224.7(187.1~258.3) | -1.060 | 0.289 |
| CKMB (ng/mL) | | 14.4(11.7~19.5) | 14.15(12.23~18.48) | 14.9(10.6~19.6) | -0.262 | 0.793 |
| CREA (μmol/L) | | 82.1(65.5~110.6) | 82.2(65.75~111.55) | 82.1(64.2~104.7) | -0.548 | 0.584 |
| EF (%) | | 67%±6% | 67%±7% | 67%±6% | 0.204 | 0.839 |
| β-HB (mmol/L) | | 29.62 (24.33~36.18) | 25.91(19.72~29.46) | 34.36(29.15~39.28) | -4.535 | 0.024 |
| IL-1β (ng/L) | | 48.14(37~59.12) | 45.95 (33.49~55.02) | 52.81(43.96~60.29) | -2.022 | 0.043 |
| IL-6 (pg/mL) | | 30.02(25.15~36.24) | 28.19(23.675~33.45) | 32.32(27.995~39.785) | -3.075 | 0.002 |
| IL-10 (pg/mL) | | 758.17±259.05 | 716.728±265.5 | 800.8±249.4 | -1.548 | 0.125 |
| TNF-α (ng/L) | | 549.3 ±178.44 | 502.7±162 | 634±157 | -4.798 | 0.096 |
| ECC (min) | | 263.6 ±6.75 | 261.67± 8.93 | 265.5±10.2 | 0.282 | 0.025 |
| AV (h) | | 70.52 ±7.24 | 77.94 ±9.1 | 65.25 ±11.21 | -1.014 | 0.016 |
| Abbreviations: ALT: alanine amino transferase, AST: aspartate transaminase, AV: assist ventilation, Asc: ascending aorta, β-HB: β-hydroxybutyrate, BNP: brain natriuretic peptide ,CHOL: cholesterol, CKMB: creatineKinase-MB, CREA: creatinine, DD: d-dimer, DM: diabetes mellitus, ECC: extracorporeal circulation, EF: ejection fraction, FiO2: fraction of inspiration O2, FLTE: false lumen thromboembolism , GLU: glucose, HBP: hypertension, hs-CRP: hypersensitive-c-reactive-protein, HDL : high-density lipoprotein , IL-1β: human Interleukin -1 beta protein, IL-6: interleukin-6, IL-10: interleukin-10, LDL: low-density lipoprotein, LDH: lacticdehydrogenase, PaO2: partial pressure of oxygen, PLT: platelet, TG: triglyceride, TNF-α: tumor necrosis factor -α, UA: uric acid, WBC: white blood cell.  Breaking location^※^: location of aortic dissection vessel rupture, including two types - Asc and Non-Asc. | | | | | |  |

**Supplementary Table S2.** Baseline characteristics of the patients (N = 90). Univariate analysis in ALI.

| Variables | | ALI (N=30) | Non-ALI (N=30) | Control (N=30) | *P*-value |
| --- | --- | --- | --- | --- | --- |
| Gender (male) | | 26(86.7%) | 23(76.7%) | 24(80%) | 0.602 |
| Age (years) | | 52.1± 10.8 | 51.9 ±12.8 | 50.8 ±11.4 | 0.545 |
| HBP | | 22(73.3%) | 19 (63.3%) | 21(70%) | 0.696 |
| DM | | 0 | 0 | 0 | 0 |
| Smoking | | 17(56.7%) | 18(60%) | 15(50%) | 0.730 |
| Drinking | | 4 (13.3%) | 2(6.7%) | 3(10%) | 0.690 |
| Break location^※^ | |  |  |  |  |
| Asc | | 8(26.7%)) | 14(46.7%) |  |  |
| Non-Asc | | 22(73.3%) | 16(53.3%) |  |  |
| FLTE | | 12(40%) | 13(43.3%) |  |  |
| PaO_2_ (mmHg) | | 74.2(60~90) | 132.5 (108~156.5) | 85(80~110) | <0.0001 |
| PaO_2_/FiO_2_ (mmHg) | | 228.3(187.5~252.4） | 401.6(328.1~505.5) | 475(430~560) | <0.0001 |
| hs-CRP (mg/L) | | 5.5(2.4~16） | 3.8(1.7~7.1) | 3(1.2~5) | 0.524 |
| UA (mol/L) | | 438.5±131.1 | 383.7±123.4 | 414.9±121.1 | 0.149 |
| WBC (/L) | | 14.9±3.4 | 14.4±4.5 | 7.2±2.2 | 0.245 |
| PLT (/L) | | 179.6±56.8 | 177.4±52.1 | 325±34.1 | 0.525 |
| DD (mg/L) | | 14.3(4.7~27.8) | 3.5(1.6~14.9) | 0.3(0~0.5) | 0.046 |
| LDL (mmol/L) | | 2.9±0.7 | 2.7±0.6 | 1.6±0.5 | 0.517 |
| HDL (mmol/L) | | 1.1±0.3 | 1.2±0.5 | 1.24±0.6 | 0.416 |
| GLU (mmol/L) | | 8.2±1.6 | 7.9±1.7 | 5.5±1.2 | 0.793 |
| BNP (pg/mL) | | 163 (89.1~275) | 108(66.3~230.1) | 62.2(35.2~100.5) | 0.357 |
| LAC (mmol/L) | | 1.4 (1.0 ~2.5) | 1.7(1.0~2.7) | 1.2(0.5~1.7) | 0.426 |
| ALT (U/L) | | 20.5 (16 ~26.4) | 17(13.6~31.9) | 35.4(9~50) | 0.448 |
| AST (U/L) | | 24.2 (21.2~31.8) | 24.3 (19.9~ 29.9) | 22(15~40) | 0.289 |
| TG (mmol/L) | | 1.0 (0.8 ~ 2.0) | 1 (0.7~1.7) | 0.93(0.6~1.7) | 0.712 |
| CHOL (mmol/L) | | 4.5±1.1 | 4.3±0.8 | 4.5±0.9 | 0.245 |
| LDH (U/L) | | 253.6 65.1 | 221.9(181.4±262.2) | 200(100~300) | 0.709 |
| CKMB (ng/mL) | | 15.9 (12.5~ 22) | 14.1 (10.5~19.5) | 2.5 (2~4.5) | 0.149 |
| CREA (μmol/L) | | 78.4(65.0~ 110.7) | 85.4 (72.8~125.3) | 90(75~115) | 0.831 |
| EF (%) | | 0.67 ± 0.06 | 0.67±0.06 | 0.68±0.07 | 0.584 |
| β-HB (mmol/L) | | 25.8 (17.6 29.5) | 34.5(30.1~40.2) | 0.211(0.042~0.263) | 0.050 |
| IL-1β (ng/L) | | 54.1(47.1~ 61.6) | 45.1(33.8~55.9) | 90.5 (85.5~ 95.3) | 0.006 |
| IL-6 (pg/mL) | | 35±10.4 | 28.3±7.4 | 35.3±5.1 | 0.012 |
| IL-10 (pg/mL) | | 812.3±273.5 | 791.8±251.5 | 26.9±15.1 | 0.205 |
| TNF-α (ng/L) | | 628.5±157.1 | 429.9±147.5 | 740±125.3 | 0.019 |
| ECC (min) | | 250.8±9.0 | 265.5±13.6 |  |  |
| AV (h) | | 86.47±12.1 | 63.25±14.1 |  |  |
| Abbreviations: ALT: alanine amino transferase, AST: aspartate transaminase, AV: assist ventilation, Asc: ascending aorta, β-HB: β-hydroxybutyrate, BNP: brain natriuretic peptide ,CHOL: cholesterol, CKMB: creatineKinase-MB, CREA: creatinine, DD: d-dimer, DM: diabetes mellitus, ECC: extracorporeal circulation, EF: ejection fraction, FiO2: fraction of inspiration O2, FLTE: false lumen thromboembolism , GLU: glucose, HBP: hypertension, hs-CRP: hypersensitive-c-reactive-protein, HDL : high-density lipoprotein , IL-1β: human Interleukin -1 beta protein, IL-6: interleukin-6, IL-10: interleukin-10, LDL: low-density lipoprotein, LDH: lacticdehydrogenase, PaO2: partial pressure of oxygen, PLT: platelet, TG: triglyceride, TNF-α: tumor necrosis factor -α, UA: uric acid, WBC: white blood cell.  Breaking location^※^: location of aortic dissection vessel rupture. | | | | |  |

**Supplementary Table 4.** Statistical analysis of different metabolites between ALI group and Control group.

| Name | VIP | Fold change | *P*-value | Mass |
| --- | --- | --- | --- | --- |
| Mannitol | 10.03882 | 227.4356718 | 1.3733E-14 | 181 / 89 |
| Galactitol | 10.20108 | 72.31496588 | 3.49666E-17 | 181 / 101 |
| Gluconic acid | 3.425354 | 22.3179161 | 2.51567E-17 | 195 / 129 |
| Didecanoylphosphatidylcholine | 0.045479 | 20.46739317 | 6.53325E-05 | 566 / 184 |
| Biliverdin | 0.006563 | 12.19834239 | 0.000305465 | 583 / 297 |
| Glucuronic acid | 0.695887 | 12.19527152 | 1.02282E-05 | 193 / 113 |
| Hexanoic acid | 0.725397 | 11.99248758 | 5.17548E-10 | 115.1 / 71.0 |
| Phenol | 0.23718 | 9.741455593 | 3.12737E-07 | 93.0 / 65.0 |
| Galacturonic acid | 1.584453 | 9.041354724 | 3.96049E-20 | 193 / 73 |
| Histidine | 1.261313 | 7.211917647 | 1.21616E-12 | 156.1 / 110.1 |
| Trehalose | 0.174098 | 6.970557736 | 0.000238998 | 365.1 / 202.9 |
| Citric acid | 0.403276 | 6.769024184 | 1.61619E-10 | 191.0 / 111.1 |
| Phenylacetylglutamine | 0.1099 | 6.321385454 | 0.001106404 | 263.1 / 145.1 |
| Quinaldic acid | 0.029921 | 6.223973989 | 0.002114393 | 174 / 156 |
| Uracil 5-carboxylate | 0.200356 | 5.751333592 | 2.50865E-06 | 155 / 68 |
| 3-Aminosalicylic acid | 0.095346 | 4.682602329 | 0.001032566 | 154.1 / 108.0 |
| trans-Aconitic acid | 0.16323 | 3.865743846 | 1.19022E-05 | 172.8 / 85 |
| Glucuronolactone | 0.804502 | 3.28747821 | 0.001789288 | 175 / 113.1 |
| Fructose 6-phosphate | 0.025873 | 3.205104277 | 0.002385959 | 259 / 97 |
| Indole-3-methyl acetate | 0.009609 | 3.084674695 | 3.81434E-08 | 190 / 130 |
| 5'-Methylthioadenosine | 0.003911 | 2.859978533 | 0.000250576 | 298 / 136.2 |
| Indole-3-lactic Acid | 0.028932 | 2.658223669 | 0.00074646 | 206.2 / 187.9 |
| Acetylcarnitine | 0.395454 | 2.601599288 | 1.32157E-08 | 204 / 85 |
| Methionine | 0.171514 | 2.562602581 | 9.03543E-08 | 150.1 / 133.0 |
| 2-Methylcitric Acid | 0.084098 | 2.529903575 | 0.021038112 | 205 / 125 |
| Adenosine monophosphate | 0.00862 | 2.522145709 | 0.012495878 | 346.1 / 97.0 |
| cis-Aconitic acid | 0.046202 | 2.488853349 | 0.005270803 | 173.0 / 85.0 |
| Adipic acid | 0.018523 | 2.478259801 | 0.001792334 | 145.0 / 82.9 |
| myo-Inositol | 0.257781 | 2.468084177 | 9.26755E-06 | 179 / 161 |
| Methylglutaric acid | 0.015789 | 2.346337629 | 0.003780484 | 145.1 / 82.9 |
| 4-Guanidinobutanoic acid | 0.464194 | 2.335101144 | 2.76115E-06 | 146 / 87 |
| Phenyllactic acid | 0.014194 | 2.285045755 | 0.004862321 | 165.1 / 119.1 |
| Glutarylcarnitine | 0.096225 | 2.210637455 | 0.01521433 | 276 / 85 |
| 5-Methoxydimethyltryptamine | 0.008102 | 2.093545073 | 0.000244518 | 219.2 / 58.2 |
| 4-Trimethylammoniobutanoic acid | 0.103985 | 2.044050397 | 1.02451E-05 | 146 / 87 |
| Glycerol-myristate | 0.012283 | 2.000064762 | 0.000831622 | 303 / 285 |
| Erythronic acid | 0.027204 | 1.949989299 | 0.035851902 | 135.0 / 75.0 |
| Methylcysteine | 0.022381 | 1.921846314 | 0.000168657 | 134.0 / 47.0 |
| Ethyl 3-indoleacetate | 0.139775 | 1.812115434 | 0.001130501 | 204 / 130 |
| Hypoxanthine | 0.099683 | 1.807286563 | 0.000824128 | 137 / 110 |
| Cysteinylglycine | 0.175853 | 1.798285515 | 8.31289E-05 | 177 / 143 |
| 3-Hydroxyisovaleric acid | 0.047324 | 1.715167474 | 0.015280734 | 117.1 / 59.0 |
| S-Adenosylhomocysteine | 0.005797 | 1.577253075 | 0.009562942 | 383.1 / 134.0 |
| Alanine | 0.433376 | 1.566822739 | 7.48483E-05 | 90.1 / 43.9 |
| 1-Oleylglycerol | 0.024168 | 1.552796505 | 0.005172999 | 357 / 247 |
| 1-Methylhistidine | 0.192005 | 1.543285088 | 0.039450041 | 170.1 / 123.9 |
| Asymmetric dimethylarginine | 0.356309 | 1.540432259 | 8.29452E-05 | 203 / 70 |
| Sarcosine | 0.460492 | 1.5329227 | 0.000140646 | 90.1 / 44.0 |
| Beta-Alanine | 0.477106 | 1.505273155 | 0.000467313 | 90.1 / 44.0 |
| Kynurenine | 0.028999 | 1.495418415 | 0.000108957 | 209.2 / 192.0 |
| Glucosamine | 0.119063 | 1.482297082 | 7.55011E-08 | 162 / 72.1 |
| Creatine | 0.121126 | 1.4662034 | 0.024884473 | 114.1 / 44.0 |
| Creatinine | 0.134914 | 1.434809659 | 0.03267278 | 114.1 / 44.0 |
| Palmitoylcarnitine | 0.109001 | 1.389776562 | 0.002681413 | 400 / 85 |
| Pregnenolone sulfate | 0.00206 | 1.377892249 | 0.001357144 | 395 / 97 |
| Asparagine | 0.195984 | 1.327814173 | 0.013181546 | 133.1 / 74.2 |
| Lactate | 1.478991 | 1.200916036 | 0.048492355 | 89.2 / 43.0 |
| Diethanolamine | 0.056778 | 0.808294158 | 0.047002664 | 106 / 88 |
| Acetylglycine | 0.167392 | 0.806545697 | 0.015305452 | 116.0 / 74.0 |
| 11Z-Eicosenoic Acid | 3.161647 | 0.798524501 | 0.0370811 | 309.2 / 309.2 |
| Serine | 0.551722 | 0.79399095 | 0.021097235 | 106.0 / 60.0 |
| N6-methyladenosine | 0.100774 | 0.785969109 | 0.003249121 | 282.1 / 150.0 |
| Pyruvic acid | 0.188223 | 0.781451468 | 0.000269407 | 87.1 / 43.1 |
| 7Z,10Z,13Z,16Z-Docosatetraenoic acid | 1.096953 | 0.777022679 | 0.017408179 | 331.3 / 331.3 |
| Valine | 0.748606 | 0.747280253 | 1.14874E-06 | 118.1 / 55.1 |
| Lithocholic acid | 0.031519 | 0.74151315 | 0.009908064 | 375.3 / 375.3 |
| Uracil | 0.434928 | 0.724702058 | 0.029053628 | 111 / 42 |
| Tryptophan | 0.493616 | 0.721463976 | 0.002532496 | 205.2 / 188.1 |
| Tyrosine | 0.700211 | 0.721260967 | 0.007791749 | 182.1 / 136.1 |
| Norvaline | 1.014653 | 0.71690052 | 1.2115E-07 | 118.1 / 72.2 |
| Alpha-aminobutyric acid | 0.352176 | 0.712103315 | 9.9436E-06 | 104.1 / 77.2 |
| Arginine | 0.415515 | 0.692556247 | 0.00679207 | 175.1 / 70.0 |
| Petroselinic acid | 0.765339 | 0.678517064 | 0.00023263 | 281.2 / 281.2 |
| Elaidic Acid | 0.780558 | 0.666308683 | 0.000108328 | 281.2 / 281.2 |
| Oleic acid | 0.628043 | 0.658112505 | 0.000146442 | 281.2 / 281.2 |
| Chenodeoxycholic acid | 0.033712 | 0.654938287 | 0.044025631 | 391.4 / 391.4 |
| Beta-Ursodeoxycholic acid | 0.032355 | 0.652886302 | 0.041106703 | 391.4 / 391.4 |
| 2-Hydroxyphenethylamine | 0.467287 | 0.646738949 | 0.01036075 | 120 / 91 |
| Palmitoleic Acid | 0.291658 | 0.626688516 | 0.004684752 | 253.2 / 253.2 |
| Phenylalanine | 6.549415 | 0.616676926 | 4.81107E-07 | 166.1 / 120.1 |
| Palmitelaidic acid | 0.408919 | 0.61206854 | 0.006000661 | 253.2 / 253.2 |
| Cholesterol sulfate | 0.647564 | 0.601839837 | 0.000416842 | 465.3 / 96.9 |
| Hyodeoxycholic acid （HDCA） | 0.018353 | 0.593709525 | 0.03277112 | 391.4 / 391.5 |
| 4Z,7Z,10Z,13Z,16Z-Docosapentaenoic Acid | 0.469554 | 0.580053035 | 4.02052E-06 | 329.2 / 329.2 |
| Leucine | 0.734131 | 0.571849769 | 2.46026E-07 | 132.1 / 86.2 |
| Alloisoleucine | 0.588598 | 0.571841378 | 2.08214E-06 | 132.1 / 86.1 |
| Isoleucine | 0.631758 | 0.563958667 | 7.15202E-07 | 132.1 / 86.2 |
| Norleucine | 1.06612 | 0.554434415 | 2.47887E-07 | 132.1 / 86.1 |
| Indole-3-acetamide | 0.003298 | 0.552441715 | 0.020679621 | 175.1 / 130.1 |
| Choline | 0.370987 | 0.532136701 | 8.24747E-09 | 104.0 / 60.1 |
| 11Z,14Z,17Z-Eicosatrienoic Acid | 1.027306 | 0.497647876 | 2.87994E-10 | 305.2 / 305.2 |
| Glycoursodeoxycholic acid | 0.030154 | 0.487882259 | 0.030156651 | 448.3 / 448.3 |
| Cortisol | 0.10394 | 0.478036333 | 0.002196831 | 363 / 327 |
| Lysine | 0.826917 | 0.457723017 | 3.36083E-06 | 147.1 / 84.0 |
| 4Z,7Z,10Z,13Z,16Z,19Z-Docosahexaenoic Acid (DHA) | 0.258621 | 0.449967784 | 1.10409E-08 | 327.2 / 327.2 |
| Trimethylamine | 0.403858 | 0.446245821 | 0.000193575 | 60.1 / 44.1 |
| 5Z,8Z,11Z,14Z,17Z-Eicosapentaenoic Acid | 0.346305 | 0.436626796 | 1.06806E-05 | 301.2 / 301.2 |
| Dihomo-gamma-linolenic acid | 0.913233 | 0.432971422 | 5.34029E-16 | 305.2 / 261.1 |
| Arachidonic acid | 0.915607 | 0.431089114 | 1.40882E-15 | 303.2 / 303.2 |
| Glutamic acid | 0.662412 | 0.389250362 | 1.042E-07 | 148.1 / 84.1 |
| Sphinganine | 0.156073 | 0.385575258 | 1.08693E-08 | 302 / 284 |
| Allantoin | 0.381959 | 0.339585613 | 2.9634E-08 | 157.0 / 42.0 |
| Aspartic acid | 0.250636 | 0.333203992 | 5.4273E-08 | 132.0 / 88.0 |
| Glycochenodeoxycholic acid | 0.031757 | 0.325506326 | 0.039011334 | 448.4 / 74.0 |
| Glycohyodeoxycholic acid | 0.096409 | 0.324072786 | 0.000232093 | 448.4 / 74.1 |
| Cortisone | 0.239661 | 0.319424272 | 4.37388E-11 | 361.1 / 163 |
| Glycodeoxycholic acid | 0.08798 | 0.314626338 | 0.000254572 | 448.4 / 73.9 |
| Erucic Acid | 0.744271 | 0.284566469 | 4.90E-08 | 337.3 / 337.3 |
| Theophylline | 0.051575 | 0.26771825 | 0.008600555 | 181.1 / 124 |
| Uridine | 1.97168 | 0.255284433 | 7.35104E-09 | 243 / 200.1 |
| Shikimic acid | 0.087248 | 0.228242755 | 0.000399325 | 173.1 / 93.0 |
| Acetylcholine | 0.02251 | 0.149577268 | 1.74758E-15 | 146.1 / 87.0 |

**Supplementary Table 5.** Statistical analysis of different metabolites between Non-ALI group and Control group.

| Name | VIP | Fold change | *P*-value | RT(s) |
| --- | --- | --- | --- | --- |
| Mannitol | 10.19154071 | 242.370547 | 7.80512E-13 | 181 / 89 |
| Galactitol | 10.12224451 | 72.8488315 | 2.26042E-12 | 181 / 101 |
| Gluconic acid | 3.295680308 | 22.92911177 | 4.92228E-15 | 195 / 129 |
| Didecanoylphosphatidylcholine | 0.041523186 | 19.10150343 | 1.3884E-05 | 566 / 184 |
| Trehalose | 0.19839379 | 17.22170461 | 0.016987239 | 365.1 / 202.9 |
| Citric acid | 0.479032245 | 11.46920249 | 2.16637E-07 | 191.0 / 111.1 |
| Phenol | 0.229853155 | 8.793514793 | 1.35602E-11 | 93.0 / 65.0 |
| Hexanoic acid | 0.529548868 | 8.67972245 | 2.46285E-07 | 115.1 / 71.0 |
| Glucuronic acid | 0.546943879 | 7.015997127 | 6.02738E-14 | 193 / 113 |
| Histidine | 1.095361693 | 6.974203984 | 1.18338E-10 | 156.1 / 110.1 |
| Galacturonic acid | 1.143827677 | 6.75989882 | 7.37337E-14 | 193 / 73 |
| Phenylacetylglutamine | 0.107578092 | 6.438849917 | 2.63753E-05 | 263.1 / 145.1 |
| Hippuric acid | 0.032869627 | 5.481105581 | 0.0227344 | 180.1 / 105.0 |
| trans-Aconitic acid | 0.190862257 | 5.465742031 | 1.80987E-06 | 172.8 / 85 |
| Uracil 5-carboxylate | 0.159543362 | 5.077269479 | 1.22579E-06 | 155 / 68 |
| Quinaldic acid | 0.026447215 | 4.910168935 | 0.000170468 | 174 / 156 |
| cis-Aconitic acid | 0.071348768 | 3.989689518 | 0.000649829 | 173.0 / 85.0 |
| Glucuronolactone | 0.806818731 | 3.74166192 | 0.01352051 | 175 / 113.1 |
| p-Cresol | 0.051704004 | 3.651261132 | 0.006485192 | 107.1 / 76.9 |
| Biliverdin | 0.004865977 | 3.591049944 | 0.047192375 | 583 / 297 |
| Fructose 6-phosphate | 0.019415257 | 2.76456248 | 0.004609823 | 259 / 97 |
| 5-Methoxydimethyltryptamine | 0.01496507 | 2.747544253 | 1.90577E-05 | 219.2 / 58.2 |
| 5'-Methylthioadenosine | 0.001947654 | 2.588380218 | 0.000646133 | 298 / 136.2 |
| Methionine | 0.15116395 | 2.582996295 | 8.58697E-08 | 150.1 / 133.0 |
| Methylcysteine | 0.020022318 | 2.446034234 | 1.61794E-05 | 134.0 / 47.0 |
| Acetylcarnitine | 0.223505343 | 2.367739258 | 3.98688E-07 | 204 / 85 |
| Adipic acid | 0.014657277 | 2.340864553 | 0.001759145 | 145.0 / 82.9 |
| myo-Inositol | 0.104644806 | 2.261447347 | 5.12646E-08 | 179 / 161 |
| Methylglutaric acid | 0.015349665 | 2.251698781 | 0.004052625 | 145.1 / 82.9 |
| 3-Aminosalicylic acid | 0.043106483 | 2.243172712 | 0.002854037 | 154.1 / 108.0 |
| Adenosine monophosphate | 0.005904316 | 2.202897469 | 0.00107538 | 346.1 / 97.0 |
| Cysteinylglycine | 0.167875834 | 2.165283593 | 0.038360891 | 177 / 143 |
| Indole-3-methyl acetate | 0.000933399 | 1.972150876 | 0.000249794 | 190 / 130 |
| Hypoxanthine | 0.215976622 | 1.948215702 | 0.004702618 | 137 / 110 |
| 4-Guanidinobutanoic acid | 0.212824405 | 1.813121278 | 6.04637E-05 | 146 / 87 |
| Sebacic acid | 0.014770569 | 1.812112474 | 0.042496296 | 201.1 / 139.1 |
| Indoxyl sulfate | 0.004952304 | 1.791101104 | 0.032354162 | 211.9 / 79.9 |
| Indole-3-lactic Acid | 0.005992998 | 1.762367103 | 0.00413539 | 206.2 / 187.9 |
| 4-Trimethylammoniobutanoic acid | 0.097135307 | 1.738219575 | 0.000166531 | 146 / 87 |
| Phenyllactic acid | 0.003462154 | 1.630559749 | 0.030876415 | 165.1 / 119.1 |
| Alanine | 0.329092631 | 1.593722494 | 0.00013317 | 90.1 / 43.9 |
| Beta-Alanine | 0.346434576 | 1.592814669 | 0.000519686 | 90.1 / 44.0 |
| Sarcosine | 0.342819099 | 1.584922258 | 0.000154529 | 90.1 / 44.0 |
| Ethyl 3-indoleacetate | 0.073622828 | 1.435641126 | 0.049910948 | 204 / 130 |
| Asymmetric dimethylarginine | 0.39360226 | 1.418245416 | 0.001546455 | 203 / 70 |
| Glucosamine | 0.118132243 | 1.410461823 | 1.19285E-06 | 162 / 72.1 |
| Kynurenine | 0.03814057 | 1.406371819 | 0.000282126 | 209.2 / 192.0 |
| Pregnenolone sulfate | 0.001837722 | 1.38957266 | 0.000185784 | 395 / 97 |
| Lactate | 1.196780648 | 1.363993358 | 0.000864297 | 89.2 / 43.0 |
| Betaine | 0.175457621 | 1.2594516 | 0.039020323 | 118.1 / 58.1 |
| Glycine | 0.352078358 | 1.239888749 | 0.039611823 | 76.0 / 30.1 |
| Acetylglycine | 0.165582024 | 0.827641464 | 0.031354112 | 116.0 / 74.0 |
| Conjugated linoleic acids | 0.706765592 | 0.821232305 | 0.034573873 | 279.2 / 279.2 |
| Linoleic acid | 0.683555394 | 0.815415422 | 0.028944888 | 279.2 / 279.2 |
| Linoelaidic acid | 0.60671195 | 0.813938396 | 0.021506367 | 279.2 / 279.2 |
| Serine | 0.593812913 | 0.810015929 | 0.015448867 | 106.0 / 60.0 |
| Picolinic acid | 0.123676872 | 0.797532604 | 0.000184753 | 124.2 / 106.1 |
| Cytosine | 2.816579238 | 0.796718495 | 0.000323672 | 112 / 94.9 |
| Thymideine-monophosphate | 0.145969931 | 0.78963613 | 0.001572101 | 321 / 195 |
| N6-methyladenosine | 0.107881551 | 0.784207086 | 0.001191447 | 282.1 / 150.0 |
| Lithocholic acid | 0.030360251 | 0.783090544 | 0.030795216 | 375.3 / 375.3 |
| Ribonic acid | 0.06341837 | 0.765554885 | 0.010078761 | 165.0 / 75.0 |
| 7Z,10Z,13Z,16Z-Docosatetraenoic acid | 1.171774111 | 0.749228355 | 0.009249866 | 331.3 / 331.3 |
| Diethanolamine | 0.062070574 | 0.733174268 | 0.009320874 | 106 / 88 |
| Uracil | 0.446223386 | 0.728339832 | 0.022244012 | 111 / 42 |
| Sabinic acid | 0.009807219 | 0.691929026 | 0.033344609 | 215 / 169 |
| Palmitoleic Acid | 0.307345785 | 0.685628318 | 0.025386602 | 253.2 / 253.2 |
| 11Z-Eicosenoic Acid | 3.462985347 | 0.683205195 | 0.001191326 | 309.2 / 309.2 |
| Palmitelaidic acid | 0.430441584 | 0.682178125 | 0.025079345 | 253.2 / 253.2 |
| 2-Hydroxyphenethylamine | 0.531482575 | 0.660364701 | 0.018089667 | 120 / 91 |
| Valine | 0.838636863 | 0.657587397 | 3.27091E-09 | 118.1 / 55.1 |
| Lysine | 0.752995395 | 0.650132022 | 0.013860278 | 147.1 / 84.0 |
| Norvaline | 1.114280664 | 0.650096551 | 2.7835E-09 | 118.1 / 72.2 |
| Tyrosine | 0.768827191 | 0.632215013 | 0.000536292 | 182.1 / 136.1 |
| Elaidic Acid | 0.829059092 | 0.629459266 | 4.71005E-05 | 281.2 / 281.2 |
| Petroselinic acid | 0.827061388 | 0.614968902 | 1.61072E-05 | 281.2 / 281.2 |
| Oleic acid | 0.677364773 | 0.610234506 | 8.10974E-06 | 281.2 / 281.2 |
| Phenylalanine | 7.362803815 | 0.591693967 | 1.1083E-06 | 166.1 / 120.1 |
| Alloisoleucine | 0.656800045 | 0.591163139 | 0.00143243 | 132.1 / 86.1 |
| Tryptophan | 0.587280274 | 0.589443786 | 6.63951E-09 | 205.2 / 188.1 |
| 4Z,7Z,10Z,13Z,16Z-Docosapentaenoic Acid | 0.497961664 | 0.581859148 | 4.21669E-05 | 329.2 / 329.2 |
| Leucine | 0.821716946 | 0.56914715 | 0.000118528 | 132.1 / 86.2 |
| Isoleucine | 0.706043845 | 0.563070744 | 0.000154544 | 132.1 / 86.2 |
| Norleucine | 1.190199399 | 0.55461399 | 8.281E-05 | 132.1 / 86.1 |
| Arginine | 0.489605562 | 0.544900247 | 5.70748E-06 | 175.1 / 70.0 |
| Cholesterol sulfate | 0.730788449 | 0.531570485 | 0.00034019 | 465.3 / 96.9 |
| Indole-3-acetamide | 0.003642413 | 0.513326953 | 0.012830215 | 175.1 / 130.1 |
| Cortisol | 0.112587607 | 0.505314651 | 0.000148937 | 363 / 327 |
| 11Z,14Z,17Z-Eicosatrienoic Acid | 1.078995322 | 0.502958271 | 1.39417E-09 | 305.2 / 305.2 |
| Allantoin | 0.390681726 | 0.463242419 | 0.001229439 | 157.0 / 42.0 |
| 5Z,8Z,11Z,14Z,17Z-Eicosapentaenoic Acid | 0.354761916 | 0.460992262 | 6.21032E-05 | 301.2 / 301.2 |
| Aspartic acid | 0.252195305 | 0.459800927 | 0.000745127 | 132.0 / 88.0 |
| Choline | 0.410407006 | 0.455204172 | 8.1544E-11 | 104.0 / 60.1 |
| Docosahexaenoic Acid (DHA) | 0.27368042 | 0.444641747 | 6.47496E-08 | 327.2 / 327.2 |
| Homocysteine thiolactone | 0.625679454 | 0.441687957 | 0.011633022 | 118 / 90 |
| Glycoursodeoxycholic acid | 0.035960481 | 0.438998291 | 0.018185397 | 448.3 / 448.3 |
| Sphinganine | 0.161500071 | 0.425220744 | 9.48526E-08 | 302 / 284 |
| Uridine | 2.039773678 | 0.414662436 | 0.004601916 | 243 / 200.1 |
| Arachidonic acid | 0.977136365 | 0.407699705 | 5.23522E-15 | 303.2 / 303.2 |
| Dihomo-gamma-linolenic acid | 0.97661884 | 0.4011255 | 2.32682E-16 | 305.2 / 261.1 |
| Trimethylamine | 0.427661005 | 0.392631942 | 6.43189E-05 | 60.1 / 44.1 |
| Glutamic acid | 0.729077109 | 0.364608485 | 6.68533E-08 | 148.1 / 84.1 |
| Taurochenodeoxycholic acid | 0.034222691 | 0.35495977 | 0.031720355 | 498.4 / 80.0 |
| Cortisone | 0.24151855 | 0.350773743 | 2.62146E-10 | 361.1 / 163 |
| Taurodeoxycholic acid | 0.039299948 | 0.338594342 | 0.031923839 | 498.4 / 80.0 |
| Quinoline | 0.009102466 | 0.333813146 | 0.031397579 | 130 / 77 |
| Erucic Acid | 0.759177156 | 0.312865953 | 5.07254E-07 | 337.3 / 337.3 |
| Glycochenodeoxycholic acid | 0.038266728 | 0.311891333 | 0.042462262 | 448.4 / 74.0 |
| Theophylline | 0.056542212 | 0.24113965 | 0.005605026 | 181.1 / 124 |
| Acetylcholine | 0.023793922 | 0.197803902 | 2.99269E-08 | 146.1 / 87.0 |
| Glycohyodeoxycholic acid | 0.112894711 | 0.181702383 | 7.71129E-06 | 448.4 / 74.1 |
| Glycodeoxycholic acid | 0.103021793 | 0.175244737 | 9.97072E-06 | 448.4 / 73.9 |

**Supplementary Table 6.** Statistical analysis of different metabolites between ALI group and Non-ALI group.

| Name | VIP | Fold change | P-value | RT(s) |
| --- | --- | --- | --- | --- |
| Biliverdin | 0.004466 | 3.396873 | 0.0293 | 583 / 297 |
| 3-Aminosalicylic acid | 0.111447 | 2.087491 | 0.037494 | 154.1 / 108.0 |
| Glycocholic acid | 0.03664 | 1.893917 | 0.048184 | 464.4 / 74.0 |
| Glycodeoxycholic acid | 0.050582 | 1.795354 | 0.013065 | 448.4 / 73.9 |
| Glycohyodeoxycholic acid | 0.055718 | 1.783536 | 0.01253 | 448.4 / 74.1 |
| Glucuronic acid | 1.548202 | 1.738209 | 0.031316 | 193 / 113 |
| Glycerol-myristate | 0.065116 | 1.631061 | 0.008328 | 303 / 285 |
| Indole-3-methyl acetate | 0.016901 | 1.564117 | 0.004951 | 190 / 130 |
| Trimethyllysine | 0.268722 | 1.510124 | 0.04227 | 189 / 84 |
| 1-Oleylglycerol | 0.05319 | 1.463845 | 0.00928 | 357 / 247 |
| Carnitine | 0.488197 | 1.394926 | 0.043094 | 162.1 / 103.1 |
| Creatinine | 0.363594 | 1.389086 | 0.04117 | 114.1 / 44.0 |
| Creatine | 0.369359 | 1.387111 | 0.041415 | 114.1 / 44.0 |
| Ornithine | 0.011157 | 1.356511 | 0.011332 | 133.1 / 70.1 |
| Galacturonic acid | 2.729082 | 1.337499 | 0.006207 | 193 / 73 |
| Ethyl 3-indoleacetate | 0.294386 | 1.262234 | 0.033788 | 204 / 130 |
| Palmitoylcarnitine | 0.207844 | 1.244644 | 0.046779 | 400 / 85 |
| 3-Dehydroshikimate | 0.142713 | 1.209879 | 0.01782 | 171 / 127 |
| Arachidic Acid | 3.877761 | 1.203145 | 0.036401 | 311.3 / 311.3 |
| Citric acid | 0.187254 | 0.590191 | 0.010614 | 191.0 / 111.1 |
| Isocitric acid | 0.018582 | 0.566928 | 0.004496 | 191.0 / 73.0 |
| Uridine 5'-diphosphate | 0.026795 | 0.44011 | 0.004452 | 403 / 111 |
| Sphingomyelin | 0.114605 | 0.293203 | 0.036158 | 731 / 184 |
